# Divergent Effect of Central Incretin Receptors Inhibition in a Rat Model of Sporadic Alzheimer’s Disease

**DOI:** 10.3390/ijms23010548

**Published:** 2022-01-04

**Authors:** Jelena Osmanovic Barilar, Ana Knezovic, Jan Homolak, Ana Babic Perhoc, Melita Salkovic-Petrisic

**Affiliations:** 1 Department of Pharmacology, School of Medicine, University of Zagreb, 10000 Zagreb, Croatia; josmanov@mef.hr (J.O.B.); jan.homolak@mef.hr (J.H.); ana.babic@mef.hr (A.B.P.); melitas@mef.hr (M.S.-P.); 2 Croatian Institute for Brain Research, School of Medicine, University of Zagreb, 10000 Zagreb, Croatia

**Keywords:** glucagon-like peptide-1, gastric inhibitory polypeptide, hippocampus, hypothalamus, Alzheimer’s disease

## Abstract

The incretin system is an emerging new field that might provide valuable contributions to the research of both the pathophysiology and therapeutic strategies in the treatment of diabetes, obesity, and neurodegenerative disorders. This study aimed to explore the roles of central glucagon-like peptide-1 (GLP-1) and gastric inhibitory polypeptide (GIP) on cell metabolism and energy in the brain, as well as on the levels of these incretins, insulin, and glucose via inhibition of the central incretin receptors following intracerebroventricular administration of the respective antagonists in healthy rats and a streptozotocin-induced rat model of sporadic Alzheimer’s disease (sAD). Chemical ablation of the central GIP receptor (GIPR) or GLP-1 receptor (GLP-1R) in healthy and diseased animals indicated a region-dependent role of incretins in brain cell energy and metabolism and central incretin-dependent modulation of peripheral hormone secretion, markedly after GIPR inhibition, as well as a dysregulation of the GLP-1 system in experimental sAD.

## 1. Introduction

Alzheimer’s disease (AD) is a progressive neurodegenerative disease closely connected to the brain’s insulin resistant state and dysregulated glucose metabolism [1]. A growing body of research shows that diabetes mellitus type 2 (T2DM) is a risk factor for AD and that drugs that can effectively treat T2DM may also elicit neuroprotective effects [1], like drugs acting on the incretin system [2,3,4].

Gastric inhibitory polypeptide (GIP) and glucagon-like peptide-1 (GLP-1) are the most important incretins for glucose regulation secreted by the gut upon food intake. As far as we know, their primary goal is to stimulate insulin secretion from pancreatic β cells [5,6]. Upon their secretion, these incretins are degraded by dipeptidyl peptidase 4 (DPP-4), which has a higher affinity towards GLP-1 than GIP. The most widely known physiological role of incretins upon their secretion is stimulation of insulin secretion by binding to the GIP receptor (GIPR) or the GLP-1 receptor (GLP-1R) on the pancreatic β cell [7]. Additionally, GLP-1 slows down gastric emptying and inhibits glucose-dependent glucagon secretion. In contrast, GIP promotes energy storage via direct actions on adipose tissue and stimulates bone formation via stimulation of osteoblast proliferation and inhibition of apoptosis [8].

Besides target sites on the periphery, these closely related incretins also have targets in the brain. GLP-1 is produced in the nucleus of the solitary tract and the caudal brainstem, which project to the hypothalamus and cortical, hypothalamic, and hippocampal nuclei [9], while opposite findings were discovered regarding GIP production in the brain [10,11,12]. GIP and GLP-1 receptors are expressed in the cerebral cortex, hippocampus, olfactory bulb, mammillary bodies, brain stem, area postrema, and cerebellum [10,13]. GLP-1 promotes satiety and GLP-1 receptor activation has been associated with weight loss in both preclinical and clinical studies [14]. However, there is more to these hormones than their action on food consumption and weight gain.

Recently, new studies showed that incretins influence several pathways in the brain, including neuroinflammation, mitochondrial function, cellular proliferation and apoptosis [15]. Deficits in cell energy function, neuronal dysfunction, and neuroinflammation are some of the pathophysiological signs of neurodegenerative disorders such as AD [16]. These features of AD can also be found in certain animal models, like the rat model, induced by intracerebroventricular streptozotocin (STZ-icv) [17,18]. Lately, incretin analogues have also been tested as possible therapeutic agents in the STZ-icv rat model of sAD [19,20], since STZ is considered a diabetogenic compound. Analogues of incretins showed a neuroprotective effect, reduction in Aβ deposition, decreased inflammatory response, enhancement of synaptic plasticity, hippocampal neurogenesis, long-term potentiation (LTP), prevention of hippocampal synapse loss and oxidative stress, and improvement of cognitive deficit in animal AD models [10,15,19,20,21].

**Figure 1:**
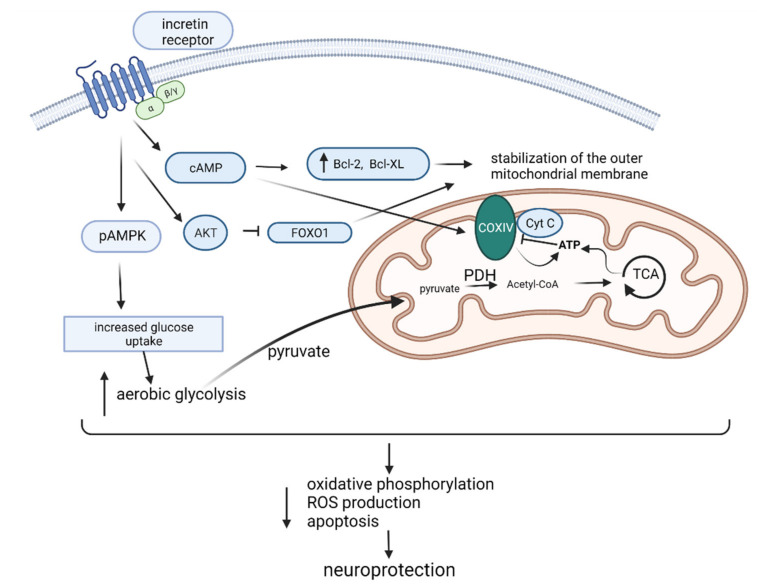
The underlying mechanism responsible for the beneficial effect of incretins in Alzheimer’s disease. Incretin receptor activation may include stabilization of the outer mitochondrial membrane, prevention of cytochrome c (CytC) efflux into the cytoplasm, and reduction of apoptosis and oxidative stress through activation of cAMP and other kinases. Together with ATP, the cAMP-dependent, protein kinase A (PKA) signaling pathway can regulate cytochrome c oxidase (COXIV) activity and mitochondrial function [22]. It can also activate AMP-activated protein kinase (AMPK), leading to increased glucose uptake by the cells in an insulin-independent manner. By increasing aerobic glycolysis, pyruvate is converted to acetyl-CoA by pyruvate dehydrogenase (PDH), which enters the citric acid cycle (TCA) to produce ATP. In this way, incretins shift the cell metabolism from oxidative phosphorylation and increased ROS production to aerobic glycolysis, leading to a neuroprotective effect. FOXO1—forkhead box protein O1; AKT—protein kinase B; Bcl—B-cell lymphoma. Figure created with BioRender.com (accessed on 27 December 2021).

The underlying mechanism responsible for the beneficial effect of GLP-1 and GIP (Figure 1) may include stabilization of the outer mitochondrial membrane, prevention of cytochrome c (CytC) efflux into the cytoplasm, and reduction of the activation of caspases 9 and 3, resulting in reduced apoptosis and oxidative stress through activation of cAMP and other kinases downstream of their signaling cascade [8,15].

A growing body of evidence also indicates that AMP-activated protein kinase (AMPK), an energy sensor, can be activated by GLP-1R agonists. It seems that GLP-1 signaling activates AMPK by phosphorylation of Thr172, leading to increased glucose uptake by the cells in an insulin-independent manner [23,24], implicating another role of incretins in brain energy homeostasis. Newer data also indicate that AMPK can increase the synthesis of GLP-1 in pancreatic β cells [25]. Both GIP and GLP-1 can influence pyruvate dehydrogenase (PDH) activity by increasing aerobic glycolysis, which in turn increases production of pyruvate, the major substrate for PDH. PDH converts pyruvate to acetyl-CoA, which enters the citric acid cycle to produce adenosine triphosphate (ATP) [26]. In this way, incretins shift the cell metabolism from oxidative phosphorylation and increase ROS production to aerobic glycolysis, leading to a neuroprotective effect [27] (Figure 1). In contrast to the aforementioned animal studies, studies in human sAD have focused on exploring the effect of GLP-1 analogues and dual GIP/GLP-1 analogues on cognition, without exploring the pathophysiological background of their beneficial effect in neurodegeneration [2,3,21]. Most experimental studies used GLP-1 and GIP antagonists to explore the physiological role of incretins on the periphery, e.g., the effect on insulin and glucagon secretion [28,29,30,31,32,33,34,35,36], but studies focusing on central inhibition in order to explore the incretin effect in the brain are few [32,37,38]. Failure of numerous drugs targeting amyloid beta and tau proteins in AD clinical trials diverted the attention to other possible drug targets. As countless studies have shown the importance of metabolic dysregulation in AD development and progression [39,40], studying brain metabolism and energy in the STZ-icv rat model of sporadic AD could lead to a better translation of preclinical data to clinical practice. As the incretin system now presents a compelling drug target (agonists in the treatment of diabetes and antagonists in the treatment of obesity) and the knowledge of its role in physiological and pathological processes in the brain are still obscure, the aim of this study was to more closely explore the role of central GLP-1 and GIP signaling inhibition on cell metabolism and energy in the brain, as well as on the peripheral levels of these incretins, insulin and glucose, by inhibiting the central incretin receptors following intracerebroventricular administration of the respective receptor antagonist. Additionally, by inhibiting the central incretin system, we explored its homeostasis and possible dysfunction in the STZ-icv rat model. The acute chemical ablation of central GIPR or GLP-1R in healthy animals and the streptozotocin-induced rat model of sAD was shown to have a region- and incretin-dependent role in the brain cell energy and metabolism, and an incretin-dependent modulation of the brain–gut axis, as well as dysregulation of these functions in experimental sAD.

## 2. Results

### 2.1. Peripheral Changes Were More Pronounced following Central Gipr Inhibition

**Figure 2:**
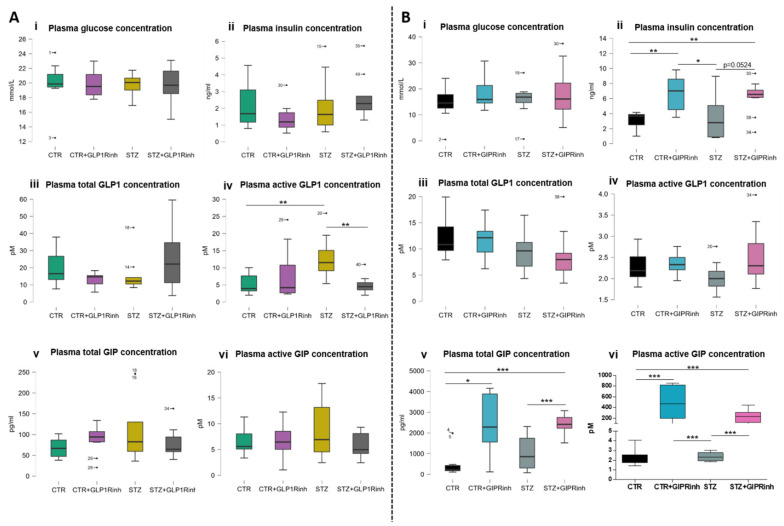
Glucose, insulin, glucagon-like peptide-1, and gastric inhibitory polypeptide plasma concentrations. One month after intracerebroventricular (icv) streptozotocin (STZ) or vehicle (CTR) administration, rats were injected icv with 85 µg/kg of glucagon-like peptide-1 receptor antagonist (exendin fragment 9-39, GLP1Rinh; experiment 1) or 85 µg/kg of gastric inhibitory polypeptide receptor antagonist (Pro3-GIP, GIPRinh; experiment 2) dissolved in saline or with saline only (CTR and STZ). Animals were sacrificed 30 min after icv administration and blood was sampled for analysis of glucose (**i**), insulin (**ii**), total (**iii**) and active (**iv**) GLP1, and total (**v**) and active (**vi**) GIP concentrations in plasma (**A**,**B**). Values are presented as boxplots with marked outliers and data analyzed by a non-parametric Kruskal–Wallis one-way ANOVA test followed by a Mann–Whitney U test (* *p* < 0.05; ** *p* < 0.01; *** *p* < 0.001).

Central inhibition of the GIPR had a stronger impact than GLP-1R inhibition on plasma levels of the measured hormones (Figure 2A,B). Both healthy and STZ-icv-treated rats demonstrated a significant increase of plasma hormone levels after GIPR inhibition—insulin (118%, *p* = 0.0016 and 73%, *p* = 0.0524), total (310.9%, *p* = 0.0343 and 137%, *p* = 0.0002) and active GIP (212-fold, *p* = 0.0004, and 96-fold, *p* < 0.0001), respectively (Figure 2B). In contrast, central GLP-1R inhibition induced no acute changes in plasma hormones in healthy rats but decreased active GLP-1 levels in the STZ-icv group (−61.3%; *p* = 0.0019) (Figure 2A(iv)). Plasma glucose concentration remained unchanged (plasma glucose concentration in Figure 2(Ai) already published in Supplementary Data S2 [41]) but the observed concentrations were quite high in each treated group due to the absence of a fasting period and systemic anesthesia. However, the effect on glucose levels could have also been masked by increased baseline glucose concentration due to icv treatment.

### 2.2. Central GLP-1R Inhibition Has a Stronger Impact on Brain Proteins Involved in Cell Energy

**Figure 3:**
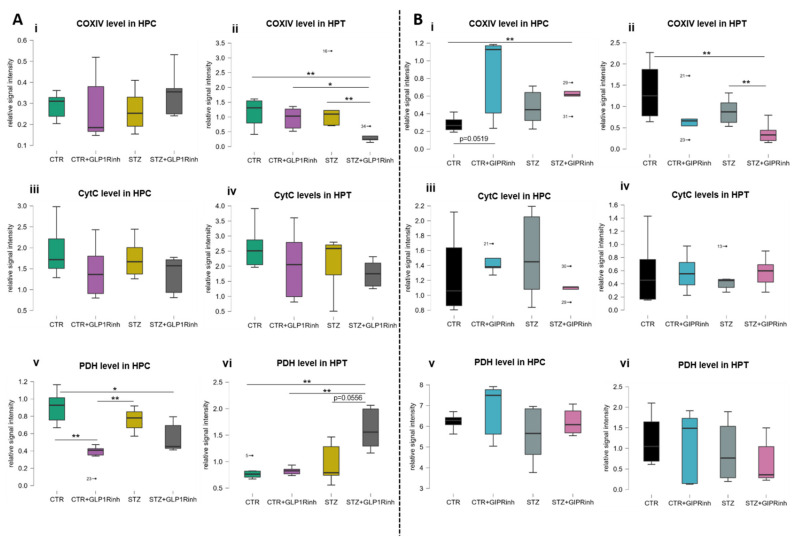
Impact of central glucagon-like peptide-1 and gastric inhibitory polypeptide inhibition on the levels of cytochrome C, cytochrome C oxidase IV, and pyruvate dehydrogenase in the hippocampus and hypothalamus. One month after intracerebroventricular (icv) streptozotocin (STZ) or vehicle (CTR) administration, rats were injected icv with 85 µg/kg of glucagon-like peptide-1 receptor antagonist (Exendin fragment 9–39, GLP1Rinh; experiment 1) or 85 µg/kg of gastric inhibitory polypeptide receptor antagonist (Pro3-GIP, GIPRinh; experiment 2) dissolved in saline or with saline only (CTR and STZ). Animals were sacrificed 30 min after icv administration and the hippocampus (HPC) and hypothalamus (HPT) were dissected, homogenized/sonicated, and protein concentration was measured. Cytochrome C (CytC; **iii**,**iv**), cytochrome C oxidase IV (COXIV; **i**,**ii**), and pyruvate dehydrogenase (PDH; **v**,**vi**) levels in the HPC and HPT were measured by Western blot analysis 30 min after central GLP1R (**A**) and GIPR (**B**) inhibition. Values are presented as boxplots with marked outliers and data analyzed by a non-parametric Kruskal–Wallis one-way ANOVA test followed by a Mann–Whitney U test (* *p* < 0.05; ** *p* < 0.01).

The levels of proteins involved in the brain cell energy status were region-dependent and more pronounced following central GLP-1R inhibition, particularly in the HPT of the STZ group compared to the controls. Both GLP-1R and GIPR inhibition decreased hypothalamic COXIV signal in the STZ group by 76.2% (*p =* 0.0079, Figure 3A(ii)) and by 57.7% (*p* = 0.0087, Figure 3B(ii)) versus the untreated STZ group, respectively, and GLP-1R inhibition decreased it by 65.5% in STZ compared to the CTR+GLP1Rinh group (Figure 3A(ii)). However, only GIPR inhibition altered the level of COXIV in the HPC by increasing it in the controls (+191%, *p =* 0.0519) (Figure 3B(i)). CytC levels remained unchanged after central GIPR and GLP-1R inhibition in both regions (Figure 3A(iii,iv),B(iii,iv)). PDH levels were altered only after GLP-1R inhibition in both regions—decreased in the HPC of controls (−60.6%, *p* = 0.0022 vs. CTR alone; Figure 3A(v)) and increased in the HPT of the STZ group both versus STZ alone (+66.9%, *p =* 0.0556) and the CTR+GLP1Rinh (+96.1%, *p =* 0.0043) group (Figure 3A(vi)). PDH levels remained unchanged following GIPR inhibition (Figure 3B(v,vi)).

### 2.3. Central Inhibition of the GIPR Has a More Pronounced Impact on Ampk Protein Levels and Activity

**Figure 4:**
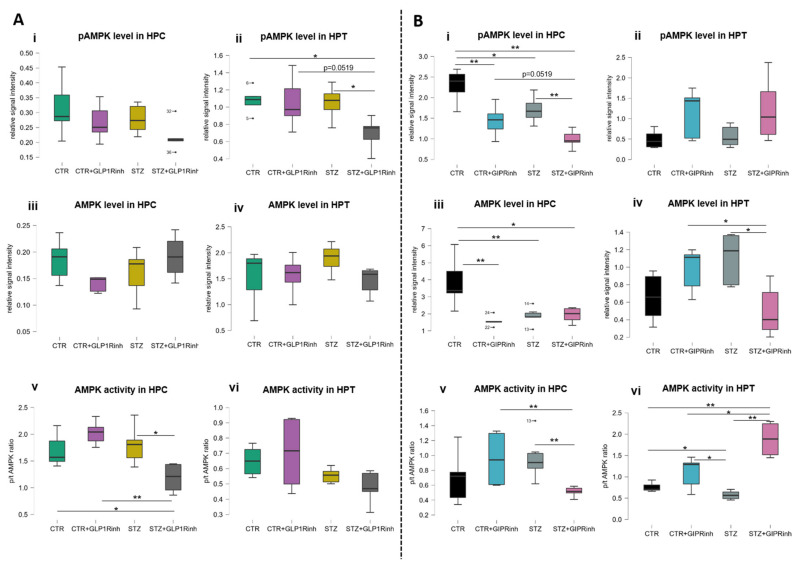
Levels and activity of AMP-activated protein kinase in the hippocampus and hypothalamus changed after central glucagon-like peptide-1 and gastric inhibitory polypeptide inhibition. One month after intracerebroventricular (icv) streptozotocin (STZ) or vehicle (CTR) administration, rats were injected icv with 85 µg/kg of glucagon-like peptide-1 receptor antagonist (Exendin fragment 9–39, GLP1Rinh; experiment 1) or 85 µg/kg of gastric inhibitory polypeptide receptor antagonist (Pro3-GIP, GIPRinh; experiment 2) dissolved in saline or with saline only (CTR and STZ). Animals were sacrificed 30 min after icv administration and the hippocampus (HPC) and hypothalamus (HPT) were dissected, homogenized/sonicated, and protein concentration was measured. Phosphorylated (Thr172) and total AMP-activated protein kinase (pAMPK (**i**,**ii**) and tAMPK (**iii**,**iv**)) levels in the HPC and HPT were measured by Western blot analysis 30 min after central GLP1R (**A**) and GIPR (**B**) inhibition. AMPK activity is expressed as a ratio between pAMPK and tAMPK (**v**,**vi**). Values are presented as boxplots with marked outliers and data analyzed by a non-parametric Kruskal–Wallis one-way ANOVA test followed by a Mann–Whitney U test (* *p* < 0.05; ** *p* < 0.01).

Following GIPR inhibition, there was a pronounced, region-dependent change in signal/activity of AMPK in the control and STZ group. The level of pAMPK was decreased in the HPC of both groups following GIPR inhibition (CTR + GIPRinh vs. CTR, −37.5%, *p* = 0.0087; STZ + GIPRinh vs. STZ, −25.9%, *p* = 0.0260; Figure 4B(i)), while the increase observed in the HPT was insignificant (Figure 4B(ii)). The levels of total AMPK after GIPR inhibition were decreased in the HPC of the controls (−59.2%, *p* = 0.0022) and in the STZ alone group compared to the CTR group (−51.2%, *p* = 0.0043) (Figure 4B(iii)), while in the HPT, a decrement following GIPR inhibition was found in the STZ group (−55% vs. STZ alone, *p* = 0.0303; −49.2% vs. CTR + GIPRinh, *p* = 0.0303) (Figure 4B(iv)). AMPK activity, assessed indirectly by the phospho/total AMPK ratio, was found decreased following GIPR inhibition in the HPC of the STZ group (−46.3%, *p* = 0.0022 vs. STZ; −46%, *p* = 0.0043 vs. CTR + GIPRinh) (Figure 4B(v)). In contrast to the HPC (no change in AMPK activity in the STZ alone vs. CTR alone groups), in the HPT, AMPK activity was found decreased in the STZ group compared to the CTR one (−24.1%, *p* = 0.0152), while after GIPR inhibition it was increased in the STZ group (+228.2% vs. STZ, *p* = 0.0043; +71.1%, *p* = 0.0159 vs. CTR + GIPRinh) (Figure 4B(vi)). The only change observed following GLP-1R inhibition was decreased pAMPK level in the HPT of the STZ group (−34% vs. STZ alone, *p* = 0.0317; −34%, *p* = 0.0519 vs. CTR + GLP1Rinh) (Figure 4A(ii)) and decreased AMPK activity in the HPC of the STZ group (−34% vs. STZ alone, *p* = 0.0381; −41.6% vs. CTR + GLP1Rinh, *p* = 0.0095) (Figure 4A(v)).

### 2.4. Central Inhibition of GLP-1R Affects the Level of the Neuronal Activity Marker C-Fos, While GIPR Inhibition Decreases cAMP

**Figure 5:**
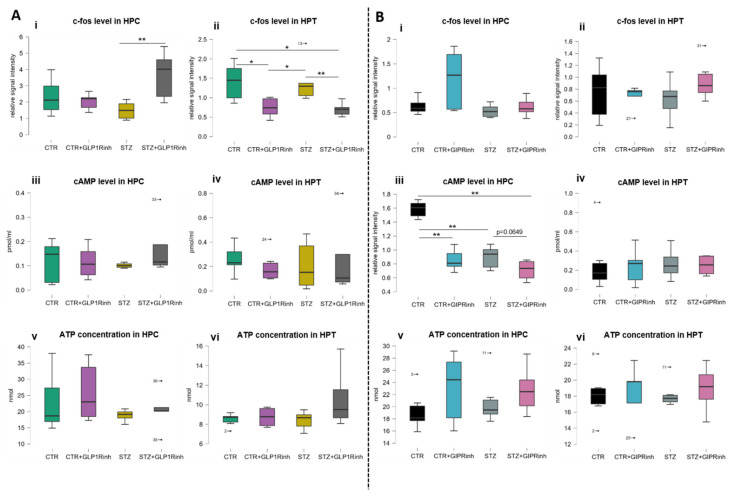
Influence of central glucagon-like peptide-1 and gastric inhibitory polypeptide inhibition on c-fos and cAMP levels and ATP concentration in the hippocampus and hypothalamus. One month after intracerebroventricular (icv) streptozotocin (STZ) or vehicle (CTR) administration, rats were injected icv with 85 µg/kg of glucagon-like peptide-1 receptor antagonist (exendin fragment 9–39, GLP1Rinh) or 85 µg/kg of gastric inhibitory polypeptide receptor antagonist (Pro3-GIP, GIPRinh) dissolved in saline or with saline only (CTR and STZ). Animals were sacrificed 30 min after icv administration and the hippocampus (HPC) and hypothalamus (HPT) were dissected, homogenized/sonicated, and protein concentration was measured. Neuronal activation, cAMP levels and ATP concentration were measured 30 min after central GLP1R (**A**) and GIPR (**B**) inhibition. Neuronal activation was measured indirectly through c-fos level by Western blot (**i**,**ii**) inhibition. cAMP levels were measured using a commercial cAMP Direct Immunoassay kit and Western blot analysis (**iii**,**iv**). ATP concentration was measured by bioluminescence assay (**v**,**vi**). Values are presented as boxplots with marked outliers and data analyzed by a non-parametric Kruskal–Wallis one-way ANOVA test followed by a Mann–Whitney U test (* *p* < 0.05; ** *p* < 0.01).

Decrements in cAMP levels were observed following GIPR inhibition in the HPC but not in the HPT, more pronounced in healthy rats (−46% vs. CTR alone, *p* = 0.0043), and to a much lesser extent in the STZ group (−18.1% vs. STZ alone, *p* = 0.0649), although the untreated STZ group had significantly decreased cAMP levels compared to the untreated controls (−43.4%, *p* = 0.0022) (Figure 5B(iii)). In contrast, the c-fos level was changed only after GLP-1R inhibition in both regions; in the HPT, decreased c-fos was observed in both the healthy (−47.1%, *p* = 0.0411) and STZ group (−49.5%, *p* = 0.0095) (Figure 5A(ii)) while in the HPC, increased c-fos was measured only in the STZ group (146.6%, *p* = 0.0087 vs. STZ alone) (Figure 5A(i)). The ATP concentration remained unchanged in both experiments in both brain regions (Figure 5A(v,vi),B(v,vi)).

## 3. Discussion

Our results indicate a substantial impact of central inhibition of incretin receptors, with consequences seen in both the periphery and in two brain regions, the HPC and the HPT, predominantly following GIPR inhibition, which led to increased active GIP plasma concentration 30 min after infusion, while GLP-1R inhibition failed to change plasma GLP-1 levels in healthy rats (Figure 2). Previous reports pointed indirectly to the central regulation of peripheral GIP release, based on decreased plasma GIP concentrations following icv insulin treatment in a canine model [42] and increased plasma GIP levels following human icv GIP treatment in nonhuman primates [43]. Given that there are no literature data on the effects of central GIPR inhibition on plasma GIP, increased plasma GIP in our research might seem contradictory to the work of Higgins et al. [43]. Higgins et al. observed that GIP treated with icv can stimulate increased plasma GIP in the absence of nutrient stimulation; however, postprandial GIP levels were not different after icv GIP infusion compared to controls [43] and animals in our research had ad libitum access to food. Other reasons for possible inconsistencies could only be speculated: (i) (Pro3)GIP, used as a GIPR antagonist in our research, acts as a weak partial agonist in mice and rat cell cultures when used with higher doses [44]; (ii) an acute block with a bolus dose of the GIPR inhibitor might activate feedback mechanisms, leading to increased peripheral GIP secretion, which could be inverted to decrease plasma GIP levels in long-term central GIPR inhibition; (iii) it could not be excluded that, to a certain extent, substantially high plasma GIP levels in our research reflect the inability of the 6A1A anti-GIP mouse IgG (used in the active GIP ELISA kit) to discriminate between the N-terminal sequence of the GIP(1-42) and [Pro^3^]-GIP. The observed increase in plasma insulin levels might provide indirect support that plasma GIP levels have indeed been increased following acute inhibition of the central GIPR, but further research should elucidate whether increased plasma insulin is an indirect or direct effect of central GIPR inhibition, or maybe both.

Although direct data on central regulation of plasma GLP-1 levels are lacking, such a possibility seems likely based on the evidence that stimulation of brain GLP-1R in rodents modulates insulin and glucagon secretion, glucose homeostasis, and corticosterone plasma levels [37,45,46,47]. Modest data on the effects of central GLP-1R inhibition indicate that during hyperglycemia, such a treatment leads to increased peripheral glucose utilization and insulin sensitization in mice [37]. We have demonstrated that, in healthy rats, acute inhibition of central GLP-1R (in contrast to GIPR inhibition) did not alter insulin, glucose, GLP-1, or GIP plasma concentrations (Figure 2A). The only change seen 30 min following GLP-1R inhibition in the STZ-icv rat model of sAD was decreased plasma concentrations of active GLP-1 (Figure 2A(iv)). A lack of this effect in control rats confirms the previously observed dysfunctional GLP-1 homeostasis in this sAD model [48]. It could not be excluded that the acute block of GLP-1 signal in the brain following central GLP-1R inhibition is compensated for by GLP-1 secretion within the brain, occurring in the nucleus of solitary tract [49]. Literature data on GIP brain secretion are inconsistent, demonstrating both its presence [12,50] and absence [10,11]. The observed discrepancy between experiments could be due to the experiments not being conducted simultaneously and due to using different rat litters. Unfortunately, this can only be circumvented by conducting the experiments at the same time with the same rat litter, which is sometimes impossible because of the large number of animals needed.

Neurodegenerative disorders, and AD in particular, are associated with T2DM-like metabolic abnormalities and insulin resistance in the brain, due to which intranasal insulin, insulin sensitizers, and GLP-1R agonists have been considered as potential therapeutic options in AD treatment [1,51]. Like diabetes, obesity also contributes to the development of AD and studies have identified several overlapping mechanisms of these disorders [52]. Basic research indicates more efficient neuroprotection of neurodegenerative and AD hallmarks with dual GLP-1/GIP agonists than GLP-1 analogues alone [53]. Additionally, GIPR and GLP-1R knockout mice showed impairment of synaptic plasticity and cognitive deficit [54,55,56]; however, in another study, GIPR knockout mice showed extended lifespan, as well as increased exploratory and decreased anxiety-based behaviors [57], indicating a divergent effect of incretins.

Efflux of inhibitors into the periphery following intracerebroventricular administration should also be acknowledged as an important limitation of the presented research. We have mentioned, in our recent research [41], a solution presented by Kanoski et al. They introduced another group that received an intraperitoneal GLP-1R inhibitor in doses that could have effluxed after icv injection and concluded that this ip treatment did not significantly attenuate intake suppression by ip delivery of agonists (the icv inhibitor antagonized peripherally administered liraglutide and exendin-4 that accessed the brain and acted directly on the CNS GLP-1R) [58]. In future research, introducing another group with peripherally administered inhibitors can be added to further distinguish central and peripheral effects.

Our results demonstrate that GIP is more important for brain cell energy and metabolism than GLP-1 and, thus, supports and provides a possible explanation for better neuroprotection of dual agonists. Direct inhibition of the brain GIPR is associated with a different pattern of changes in the HPC compared to the HPT, seen as the opposite direction of changes of the proteins involved in metabolism and energy (COXIV and AMPK, Figure 3B(i,ii) and Figure 4B). AMPK acts as a sensor of cellular energy and is activated in response to falling energy charge to stimulate energy production via catabolic pathways, while decreasing nonessential energy-consuming pathways to restore cellular energy stores [59]. Changes in AMPK homeostasis were predominantly seen following GIPR inhibition, demonstrating a region- and group-dependent effect, with AMPK activity found decreased in the STZ group in the HPC and increased in the HPT, while in controls, decreased protein levels of phospho and total AMPK (but not the activity) were found in the HPC only. Central GLP-1R inhibition decreased AMPK activity in the HPC only in the STZ group. To date, there are no data on the effect of central GIPR modulation on AMPK levels and activity in the brain. Given that GIP and GLP-1 are involved in glucose homeostasis, further research on the relationship between hypothalamic AMPK (considered as a regulator of the whole-body energy balance) and GIP/GLP-1 signaling could give insight into possible new therapeutic approaches for the treatment of obesity, diabetes, or neurodegenerative disorders. Unchanged ATP concentrations in the HPC (Figure 5A(v),B(v)) and HPT (Figure 5A(vi),B(vi)) could be due to the activation of compensatory mechanisms, since it was measured 30 min after inhibition and the ATP turnover rate is high.

Recently, novel therapeutics targeting GIP signaling, GIP antagonists, and dual GIPR and GLP-1R agonists have been regaining interest due to GIP regulation of energy homeostasis in the brain [10]. Mitochondrial COX is the primary site of cellular oxygen consumption and is essential for aerobic energy generation in the form of ATP [60]. The opposite pattern of protein levels involved in energy in the HPC and HPT after GIPR inhibition indicates a divergent action of GIP in these regions. Possible cell energy deprivation in the HPT (presented as decrement of COXIV; Figure 3B(ii); and increment of AMPK activity; Figure 4B(vi)) can lead to increased peripheral secretion of GIP and insulin, and opposite changes of protein levels (presented as COXIV level increment; Figure 3B(i); and AMPK level decrement; Figure 4B(iii)) found in the HPC can give insight into the possible involvement of GIPR signaling in cognition. Further research is needed to elucidate the action of central GIPR inhibition on learning and memory.

The majority of changes seen after acute central GLP-1R inhibition were seen in animals that were administered STZ-icv 1 month before, suggesting that STZ induces an imbalance in the central GLP-1 system. As previously reported, the therapeutic window for the efficiency of different drug treatments is up to 3 months after STZ-icv administration, a time point when cognitive decline in this model enters into the dose-dependent progressive and irreversible phase, while 1 month after STZ-icv treatment, some reversibility of the pathology could still be observed [61,62]. How the GLP1 system is involved in STZ-induced pathophysiological changes or development of AD is still not clear yet and needs further elucidation.

After central GLP1R inhibition, there was no change in cAMP levels in the HPC (Figure 5A(iii)) and HPT (Figure 5A(iv)), suggesting possible compensation through activation of GIPR signaling or some other signaling pathway that activates adenylate cyclase. Further research of specific receptor activation is needed to elucidate whether there is a compensatory response. In contrast, central inhibition of GIPR decreased cAMP levels in the HPC, while it had little impact on already decreased levels in STZ-icv treated rats (Figure 5B(iii)), indicating incretin resistance in the brain of this sAD animal model, as was seen in our previous research (STZ-icv also reduced the levels of GLP-1R in the HPC) [63]. Interestingly, plasma levels of insulin and total and active GIP were found increased after GIPR inhibition in the STZ-icv rat model, as well as in controls (Figure 2B), suggesting that the HPC does not regulate secretion of the measured hormones.

Most of the research regarding the GLP1 system in AD is based on testing GLP1 agonists or DPPIV inhibitors as potential neuroprotective agents, which could normalize insulin sensitivity in the brain [64]. The potential involvement of GLP1 signaling in the pathogenesis of human sAD has not been fully explored yet and needs further evaluation of its possible involvement in neurodegeneration. In pancreatic β-cells, chronic stimulation of the GLP-1R increases glycolysis and ATP production through transcriptional activation and expression of glycolytic genes, and inhibition of the PI3K/mTOR pathway abolishes such an effect [65]. These findings are in concordance with a decreased level of PDH in the HPC after GLP-1R inhibition, suggesting decreased glucose metabolism. If GLP-1R inhibition decreases the import of glucose, PDH activity is downregulated to limit the use of glucose by oxidative phosphorylation [26]. In contrast to the results found in the HPC, PDH levels in the HPT were increased in STZ rats, in compliance with decreased COXIV levels after GLP-1R inhibition. Whether an increased PDH level is a compensatory effect due to decreased COXIV, or vice versa, remains to be explored. In contrast, GIPR inhibition did not alter PDH levels at all, further suggesting different actions of GIP and GLP-1 in the brain (Figure 3B(v,vi)). In line with these results, only GLP-1R inhibition influenced neuronal activation, increasing it in the HPC and decreasing it in the HPT.

Due to highly increased GIP levels in plasma after acute central GIPR inhibition and the unchanged plasma GLP-1 levels after central GLP-1R inhibition, we can speculate that the CNS is more dependent on the production of GIP at the periphery than GLP-1, but there is also a possibility that the GIPR was more inhibited than the GLP1R, hence a more pronounced response. Based on literature data [66], it seems more likely that a signal to the periphery for the elevation of GIP secretion might originate from the hypothalamus than from the hippocampus, but further research is needed to confirm this hypothesis. Changes observed after GLP-1R inhibition mostly concern STZ-icv rats, and to a much lesser extent healthy controls, further supporting our previously published data on dysfunctional GLP-1 signaling in the STZ-icv rat model of sAD and involvement of the brain–gut GLP-1 axis in the maintenance of redox balance in the upper small intestine [41]. Data presented here indicate that GIPR and GLP-1R inhibition, respectively, have an opposite effect on the level of proteins involved in metabolism and energy balance regulation in the hippocampus and hypothalamus. Brain incretin signaling has emerged as a new field that might provide valuable contributions to the research of both the pathophysiology and, accordingly, novel therapeutic strategies in the treatment of diabetes, obesity, aging, and related neurodegenerative disorders.

## 4. Materials and Methods

### 4.1. Animals

Adult (3-month-old) male Wistar rats weighing 250–350 g (Department of Pharmacology, University of Zagreb School of Medicine) were used in the experiment. All animals were housed in cages (2–3 rats per cage) in the animal facility at the department, kept on standardized food pellets and water ad libitum, and maintained under a 12/12 h light/dark cycle.

### 4.2. Ethics

The experiments were carried out in compliance with current institutional (University of Zagreb School of Medicine), national (Animal Protection Act, NN 102/17), and international (Directive 2010/63/EU) guidelines on the use of experimental animals. The national regulatory body, the Croatian Ministry of Agriculture, approved the experiments (license number EP 186/2018).

### 4.3. Materials

Streptozotocin, exendin fragment 9–39, PhosSTOP phosphatase inhibitor tablets, and protease inhibitor cocktail were purchased from Sigma-Aldrich (St. Louis, MO, USA). [Pro3]-GIP (Rat) was purchased from Tocris (Abingdon, United Kingdom). The glucose measuring kit (Greiner Diagnostic Glucose GOD-PAP) was acquired from Dijagnostika (Sisak, Croatia). The chemiluminescent Western blot detection kit (SuperSignal West Femto Chemiluminescent Substrate) and ATP determination kit were from Thermo Scientific (Rockford, IL, USA). The ELISA Kit for rat/mouse insulin, GLP1, and GIP total ELISA kits, and high-sensitivity GLP1 Active ELISA kit were acquired from Merck Millipore (Billerica, MA, USA). Polyclonal anti-c-Fos antibody was purchased from Abcam (Cambridge, UK). TGX FastCast Acrylamide Solution was purchased from Bio-Rad (Hercules, CA, USA). Anti-phospho-AMPKα (Thr172), anti-AMPKα, anti-cytochrome c oxidase subunit 4 (COXIV), anti-cytochrome c (CytC), anti-pyruvate dehydrogenase (PDH), anti-mouse IgG HRP-linked antibody, and anti-rabbit IgG HRP-linked antibody were acquired from CellSignaling (Beverly, MA, USA). cAMP Direct Immunoassay Kit was purchased from BioVision (Milpitas, CA, USA). Anti-cAMP antibody (Elabscience, Houston, TX, USA) was purchased from antibodies-online. Mouse GIP active form, high sensitivity, ELISA kit was purchased from Tecan IBL International (Hamburg, Germany).

### 4.4. Experimental Design

**Figure 6:**
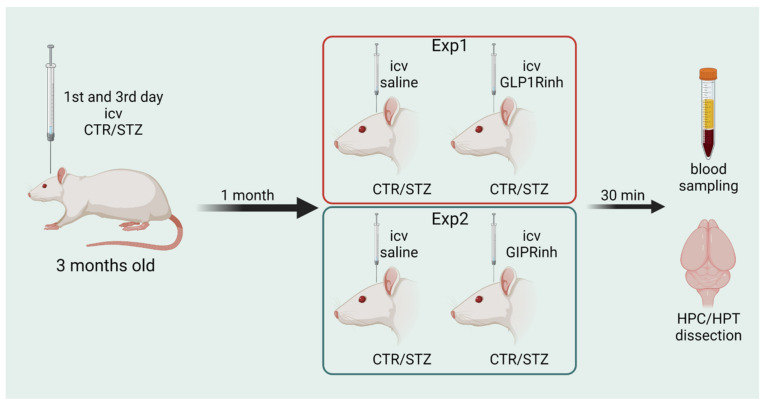
Experimental design. Two experiments with 4 groups each were conducted with the same procedure. Three-month-old male Wistar rats were intracerebroventricularly (icv) injected with streptozotocin (STZ/3 mg/kg) or vehicle only (controls/CTR) on days 1 and 3. In experiment 1 (Exp1) rats were randomly divided into four groups. Two groups (CTR + GLP1Rinh and STZ + GLP1Rinh) received icv 85 µg/kg of glucagon-like peptide-1 receptor antagonist (exendin fragment 9–39, GLP1Rinh) dissolved in saline. In experiment 2 (Exp2), two groups (CTR + GIPRinh and STZ + GIPRinh) received icv 85 µg/kg of gastric inhibitory polypeptide receptor antagonist (Pro3-GIP, GIPRinh) dissolved in saline. The other groups in both experiments (CTR and STZ) received saline only (bilaterally 2 µL/ventricle). All animals were sacrificed 30 min after icv. Blood was sampled and brain was removed and the hippocampus (HPC) and hypothalamus (HPT) were dissected out for further analysis. Figure created with BioRender.com (accessed on 21 December 2021).

Streptozotocin treatment. Two experiments with 4 groups each were conducted with the same procedure. Three-month-old male Wistar rats were subjected to general anesthesia (ketamine 70 mg/kg; 7 mg/kg xylazine), followed by intracerebroventricular (coordinates: AP: −1.5 mm; ML: ±1.5 mm; DV: +4 mm) injection of STZ (3 mg/kg, dissolved in 0.05 M citrate buffer, pH 4.5, bilaterally 2 µL/ventricle, split into two doses administered on day 1 and 3), according to the procedure first described by Noble et al. [67] and used in our previous experiments [61,68]. Control (CTR) animals were given an equal volume of vehicle icv by the same procedure (Figure 6). Conductors of the experiments were not blinded to drug/vehicle treatment.

Experiment 1: GLP1Rinh treatment. One month after STZ/citrate buffer administration, control and STZ-treated rats were randomly divided in four groups. Two groups (CTR+GLP1Rinh and STZ+GLP1Rinh) received icv 85 µg/kg (25.23 mmol/kg) of the competitive GLP-1 receptor antagonist (exendin fragment 9–39) dissolved in saline (bilaterally 2 µL/ventricle), administration protocol based on our preliminary experiment demonstrating reduced neuronal and metabolic activity after icv application of 85 µg/kg, but not after 60 and 125 µg/kg of exendin fragment 9–39 [69] and based on literature data [58,70,71]. The other 2 groups (CTR and STZ) received saline only (bilaterally 2 µL/ventricle). All animals were sacrificed 30 min after icv administration. There were 10 animals per group, except 9 animals in the STZ+GLP1Rinh group (Figure 6).

Experiment 2: GIPRinh treatment. One month after STZ/citrate buffer administration, control and STZ-treated rats were randomly divided in four groups. Two groups (CTR + GIPRinh and STZ + GIPRinh) received icv 85 µg/kg (12.19 mmol/kg) of GIP receptor antagonist (Pro3-GIP) dissolved in saline (bilaterally 2 µL/ventricle). The other 2 groups (CTR and STZ) received saline only (bilaterally 2 µL/ventricle). All animals were sacrificed 30 min after icv administration [44]. There were 10 animals per group, except 8 animals in the CTR + GIPRinh group (Figure 5).

### 4.5. Tissue Preparation and Blood Sampling

Sacrification was performed under deep general anesthesia with thiopental and diazepam (70 mg/kg and 7 mg/kg) without a prior overnight fasting period. Blood was sampled from the retro-orbital sinus of each animal. Six or five animals per group were decapitated, after which brains were quickly removed, with the hippocampus (HPC) and hypothalamus (HPT) dissected out and frozen in liquid nitrogen. The rest of the animals (3–4 per group) underwent fixative perfusion with 4% paraformaldehyde, their brains were removed and stored for future analysis. The fresh frozen brain tissue samples were sonicated in lysis buffer containing 10mM HEPES, 1mM EDTA, 100 mM KCl, 1% Triton X-100, pH 7,5, protease and phosphatase inhibitors. Homogenates were centrifuged at 13,000 rpm for 10 min at 4 °C and the supernatant was stored. Protein concentration was measured by Lowry protein assay.

### 4.6. Biochemical Analysis

Plasma glucose, insulin, GLP-1 total, GIP total, GLP-1 active, GIP active measurements, and plasma hormone levels were measured using commercial kits by adhering to the manufacturers’ protocols. Absorbance was measured using a microplate reader (Tecan Trading AG, Switzerland) and measurement of relative light units was done in a luminometer, the GloMax microplate reader (Promega, Madison, WI, USA).

ATP and cAMP measurements. Quantitative determination of ATP and cAMP concentration in tissue homogenates (HPC and HPT) was done using commercial kits by adhering to the manufacturer’s protocols.

Western blot analysis. Equal amounts of total protein (35 µg per sample) in the HPT and HPC were separated by SDS-PAGE using TGX Stain-Free 12% gels (gels were visualized using ChemiDoc Imaging Systems, Bio-rad, USA) and transferred to nitrocellulose membranes using the Trans-Blot Turbo Transfer System (Bio-rad). The membranes were blocked for 1h at RT in 5% non-fat milk, added to a low-salt washing buffer (LSWB). The blocked membranes were incubated with primary antibodies (anti-CytC, anti-COXIV, anti-PDH, anti-cAMP, anti-c-fos, anti-phospho-AMPK, or anti-total-AMPK) overnight at 4 °C. After incubation, the membranes were washed with LSWB and incubated for 1 h at RT with a secondary antibody solution (anti-rabbit or anti-mouse). After washing, signals were captured and visualized using a chemiluminescence Western blotting detection reagent with a MicroChemi video camera system (DNR Bio-Imaging Systems). Proteins were analyzed using the ImageJ software and blots (Supplementary Data S1) were normalized to total protein signal of UV-transilluminated gels.

### 4.7. Statistics

Data (Supplementary Data S1) are presented as boxplots with labeled outliers (each label represents the animal ID listed in Supplementary Data S1) with the significance of between-group differences in all analyses tested by two-tailed Kruskal–Wallis one-way ANOVA analysis of variance, followed by Mann–Whitney U-test, with *p* < 0.05 considered statistically significant using GraphPad Prism 5 and JASP version 0.15 statistical software.

## Data Availability

The data presented in this study are available in Supplementary Data S1.

## Supplementary Materials

The following supporting information can be downloaded at: www.mdpi.com/article/10.3390/ijms23010548/s1.

## References

[1] Kellar D., Craft S. (2020). Brain insulin resistance in Alzheimer’s disease and related disorders: Mechanisms and therapeutic approaches. Lancet Neurol..

[2] Femminella G.D., Frangou E., Love S.B., Busza G., Holmes C., Ritchie C., Lawrence R., McFarlane B., Tadros G., Ridha B.H. (2019). Evaluating the effects of the novel GLP-1 analogue liraglutide in Alzheimer’s disease: Study protocol for a randomised controlled trial (ELAD study). Trials.

[3] Gejl M., Gjedde A., Egefjord L., Møller A., Hansen S.B., Vang K., Rodell A., Brændgaard H., Gottrup H., Schacht A. (2016). In Alzheimer’s disease, 6-month treatment with GLP-1 analog prevents decline of brain glucose metabolism: Randomized, placebo-controlled, double-blind clinical trial. Front. Aging Neurosci..

[4] Hölscher C. (2014). Drugs developed for treatment of diabetes show protective effects in Alzheimer’s and Parkinson’s diseases. Sheng Li Xue Bao.

[5] Sandoval D.A., D’Alessio D.A. (2015). Physiology of Proglucagon Peptides: Role of Glucagon and GLP-1 in Health and Disease. Physiol. Rev..

[6] Yabe D., Seino Y. (2011). Two incretin hormones GLP-1 and GIP: Comparison of their actions in insulin secretion and β cell preservation. Prog. Biophys. Mol. Biol..

[7] Seino Y., Fukushima M., Yabe D. (2010). GIP and GLP-1, the two incretin hormones: Similarities and differences. J. Diabetes Investig..

[8] Baggio L.L., Drucker D.J. (2007). Biology of Incretins: GLP-1 and GIP. Gastroenterology.

[9] Rinaman L. (2010). Ascending projections from the caudal visceral nucleus of the solitary tract to brain regions involved in food intake and energy expenditure. Brain Res..

[10] Adriaenssens A.E., Gribble F.M., Reimann F. (2020). The glucose-dependent insulinotropic polypeptide signaling axis in the central nervous system. Peptides.

[11] Kaneko K., Fu Y., Lin H.Y., Cordonier E.L., Mo Q., Gao Y., Yao T., Naylor J., Howard V., Saito K. (2019). Gut-derived GIP activates central Rap1 to impair neural leptin sensitivity during overnutrition. J. Clin. Investig..

[12] Tseng C.C., Jarboe L.A., Landau S.B., Williams E.K., Wolfe M.M. (1993). Glucose-dependent insulinotropic peptide: Structure of the precursor and tissue-specific expression in rat. Proc. Natl. Acad. Sci. USA.

[13] Gu G., Roland B., Tomaselli K., Dolman C.S., Lowe C., Heilig J.S. (2013). Glucagon-like peptide-1 in the rat brain: Distribution of expression and functional implication. J. Comp. Neurol..

[14] Gallwitz B. (2009). Preclinical and clinical data on extraglycemic effects of GLP-1 receptor agonists. Rev. Diabet. Stud..

[15] Athauda D., Foltynie T. (2016). The glucagon-like peptide 1 (GLP) receptor as a therapeutic target in Parkinson’s disease: Mechanisms of action. Drug Discov. Today.

[16] Yin F., Sancheti H., Patil I., Cadenas E. (2016). Energy metabolism and inflammation in brain aging and Alzheimer’s disease. Free Radic. Biol. Med..

[17] Salkovic-Petrisic M., Hoyer S. (2007). Central insulin resistance as a trigger for sporadic Alzheimer-like pathology: An experimental approach. J. Neural Transm. Suppl..

[18] Correia S.C., Santos R.X., Perry G., Zhu X., IMoreira P.I., Smith M.A. (2011). Insulin-resistant brain state: The culprit in sporadic Alzheimer’s disease?. Ageing Res. Rev..

[19] Li C., Liu W., Li X., Zhang Z., Qi H., Liu S., Yan N., Xing Y., Hölscher C., Wang Z. (2020). The novel GLP-1/GIP analogue DA5-CH reduces tau phosphorylation and normalizes theta rhythm in the icv. STZ rat model of AD. Brain Behav..

[20] Paladugu L., Gharaibeh A., Kolli N., Learman C., Hall T.C., Li L., Rossignol J., Maiti P., Dunbar G.L. (2021). Liraglutide has anti-inflammatory and anti-amyloid properties in streptozotocin-induced and 5xFAD mouse models of alzheimer’s disease. Int. J. Mol. Sci..

[21] Hölscher C. (2018). Novel dual GLP-1/GIP receptor agonists show neuroprotective effects in Alzheimer’s and Parkinson’s disease models. Neuropharmacology.

[22] Valsecchi F., Ramos-Espiritu L.S., Buck J., Levin L.R., Manfredi G. (2013). cAMP and mitochondria. Physiology.

[23] Andreozzi F., Raciti G.A., Nigro C., Mannino G.C., Procopio T., Davalli A.M., Beguinot F., Sesti G., Miele C., Folli F. (2016). The GLP-1 receptor agonists exenatide and liraglutide activate Glucose transport by an AMPK-dependent mechanism. J. Transl. Med..

[24] Stojakovic A., Trushin S., Sheu A., Khalili L., Chang S.Y., Li X., Christensen T., Salisbury J.L., Geroux R.E., Gateno B. (2021). Partial inhibition of mitochondrial complex I ameliorates Alzheimer’s disease pathology and cognition in APP/PS1 female mice. Commun. Biol..

[25] Jiang S., Zhai H., Li D., Huang J., Zhang H., Li Z., Zhang W., Xu G. (2016). AMPK-dependent regulation of GLP1 expression in L-like cells. J. Mol. Endocrinol..

[26] Milne J.L.S. (2013). Structure and Regulation of Pyruvate Dehydrogenases. Encyclopedia of Biological Chemistry: Second Edition.

[27] Zheng J., Xie Y., Ren L., Qi L., Wu L., Pan X., Zhou J., Chen Z., Liu L. (2021). GLP-1 improves the supportive ability of astrocytes to neurons by promoting aerobic glycolysis in Alzheimer’s disease. Mol. Metab..

[28] Gasbjerg L.S., Helsted M.M., Hartmann B., Sparre-Ulrich A.H., Veedfald S., Stensen S., Lanng A.R., Bergmann N.C., Christensen M.B., Vilsbøll T. (2020). GIP and GLP-1 Receptor Antagonism During a Meal in Healthy Individuals. J. Clin. Endocrinol. Metab..

[29] Gasbjerg L.S., Bergmann N.C., Stensen S., Christensen M.B., Rosenkilde M.M., Holst J.J., Nauck M., Knop F.K. (2020). Evaluation of the incretin effect in humans using GIP and GLP-1 receptor antagonists. Peptides.

[30] Tseng C.C., Zhang X.Y., Wolfe M.M. (1999). Effect of GIP and GLP-1 antagonists on insulin release in the rat. Am. J. Physiol. Endocrinol. Metab..

[31] Gault V.A., O’Harte F.P.M., Harriott P., Mooney M.H., Green B.D., Flatt P.R. (2003). Effects of the novel (Pro3)GIP antagonist and exendin(9-39)amide on GIP- and GLP-1-induced cyclic AMP generation, insulin secretion and postprandial insulin release in obese diabetic (ob/ob) mice: Evidence that GIP is the major physiological incretin. Diabetologia.

[32] Turton M.D., O’Shea D., Gunn I., Beak S.A., Edwards C.M.B., Meeran K., Choi S.J., Taylor G.M., Heath M.M., Lambert P.D. (1996). A role for glucagon-like peptide-1 in the central regulation of feeding. Nature.

[33] Gault V.A., McClean P.L., Cassidy R.S., Irwin N., Flatt P.R. (2007). Chemical gastric inhibitory polypeptide receptor antagonism protects against obesity, insulin resistance, glucose intolerance and associated disturbances in mice fed high-fat and cafeteria diets. Diabetologia.

[34] Cassidy R.S., Irwin N., Flatt P.R. (2008). Effects of gastric inhibitory polypeptide (GIP) and related analogues on glucagon release at normo- and hyperglycaemia in Wistar rats and isolated islets. Biol. Chem..

[35] McClean P.L., Irwin N., Cassidy R.S., Holst J.J., Gault V.A., Flatt P.R. (2007). GIP receptor antagonism reverses obesity, insulin resistance, and associated metabolic disturbances induced in mice by prolonged consumption of high-fat diet. Am. J. Physiol. Endocrinol. Metab..

[36] Calabria A.C., Li C., Gallagher P.R., Stanley C.A., De León D.D. (2012). GLP-1 Receptor Antagonist Exendin-(9-39) Elevates Fasting Blood Glucose Levels in Congenital Hyperinsulinism Owing to Inactivating Mutations in the ATP-Sensitive K+ Channel. Diabetes.

[37] Knauf C., Cani P.D., Perrin C., Iglesias M.A., Maury J.F., Bernard E., Benhamed F., Grémeaux T., Drucker D.J., Kahn C.R. (2005). Brain glucagon-like peptide-1 increases insulin secretion and muscle insulin resistance to favor hepatic glycogen storage. J. Clin. Investig..

[38] De Jonghe B.C., Holland R.A., Olivos D.R., Rupprecht L.E., Kanoski S.E., Hayes M.R. (2016). Hindbrain GLP-1 receptor mediation of cisplatin-induced anorexia and nausea. Physiol. Behav..

[39] Cai H., Cong W., Ji S., Rothman S., Maudsley S., Martin B. (2012). Metabolic Dysfunction in Alzheimers Disease and Related Neurodegenerative Disorders. Curr. Alzheimer Res..

[40] Yan X., Hu Y., Wang B., Wang S., Zhang X. (2020). Metabolic Dysregulation Contributes to the Progression of Alzheimer’s Disease. Front. Neurosci..

[41] Homolak J., Perhoc A.B., Knezovic A., Barilar J.O., Salkovic-Petrisic M. (2021). Failure of the brain glucagon-like peptide-1-mediated control of intestinal redox homeostasis in a rat model of sporadic alzheimer’s disease. Antioxidants.

[42] Yavropoulou M.P., Kotsa K., Anastasiou O., O’Dorisio T.M., Pappas T.N., Yovos J.G. (2009). Effect of intracerebroventricular infusion of insulin on glucose-dependent insulinotropic peptide in dogs. Neurosci. Lett..

[43] Higgins P.B., Shade R.E., Rodríguez-Sánchez I.P., Garcia-Forey M., Tejero M.E., Voruganti V.S., Cole S.A., Comuzzie A.G., Folli F. (2016). CNS Control of Metabolism: Central GIP signaling stimulates peripheral GIP release and promotes insulin and pancreatic polypeptide secretion in nonhuman primates. Am. J. Physiol. Endocrinol. Metab..

[44] Sparre-Ulrich A.H., Hansen L.S., Svendsen B., Christensen M., Knop F.K., Hartmann B., Holst J.J., Rosenkilde M.M. (2016). Species-specific action of (Pro3)GIP—A full agonist at human GIP receptors, but a partial agonist and competitive antagonist at rat and mouse GIP receptors. Br. J. Pharmacol..

[45] Jessen L., Smith E.P., Ulrich-Lai Y., Herman J.P., Seeley R.J., Sandoval D., D’Alessio D. (2017). Central Nervous System GLP-1 Receptors Regulate Islet Hormone Secretion and Glucose Homeostasis in Male Rats. Endocrinology.

[46] Sandoval D.A., Bagnol D., Woods S.C., D’Alessio D.A., Seeley R.J. (2008). Arcuate glucagon-like peptide 1 receptors regulate glucose homeostasis but not food intake. Diabetes.

[47] Tudurí E., Beiroa D., Porteiro B., López M., Diéguez C., Nogueiras R. (2015). Acute but not chronic activation of brain glucagon-like peptide-1 receptors enhances glucose-stimulated insulin secretion in mice. Diabetes Obes. Metab..

[48] Knezovic A., Osmanovic Barilar J., Babic A., Bagaric R., Farkas V., Riederer P., Salkovic-Petrisic M. (2018). Glucagon-like peptide-1 mediates effects of oral galactose in streptozotocin-induced rat model of sporadic Alzheimer’s disease. Neuropharmacology.

[49] Vrang N., Hansen M., Larsen P.J., Tang-Christensen M. (2007). Characterization of brainstem preproglucagon projections to the paraventricular and dorsomedial hypothalamic nuclei. Brain Res..

[50] Nyberg J., Anderson M.F., Meister B., Alborn A.M., Ström A.K., Brederlau A., Illerskog A.C., Nilsson O., Kieffer T.J., Hietala M.A. (2005). Glucose-dependent insulinotropic polypeptide is expressed in adult hippocampus and induces progenitor cell proliferation. J. Neurosci..

[51] Watson G.S., Craft S. (2003). The role of insulin resistance in the pathogenesis of Alzheimer’s disease: Implications for treatment. CNS Drugs.

[52] Pugazhenthi S., Qin L., Reddy P.H. (2017). Common neurodegenerative pathways in obesity, diabetes, and Alzheimer’s disease. Biochim. Biophys. Acta Mol. Basis Dis..

[53] Ji C., Xue G.-F., Li G., Li D., Hölscher C. (2016). Neuroprotective effects of glucose-dependent insulinotropic polypeptide in Alzheimer’s disease. Rev. Neurosci..

[54] Abbas T., Faivre E., Hölscher C. (2009). Impairment of synaptic plasticity and memory formation in GLP-1 receptor KO mice: Interaction between type 2 diabetes and Alzheimer’s disease. Behav. Brain Res..

[55] Faivre E., Gault V.A., Thorens B., Hölscher C. (2011). Glucose-dependent insulinotropic polypeptide receptor knockout mice are impaired in learning, synaptic plasticity, and neurogenesis. J. Neurophysiol..

[56] Lennox R.R., Moffett C., Porter D.W., Irwin N., Gault V.A., Flatt P.R. (2015). Effects of glucose-dependent insulinotropic polypeptide receptor knockout and a high-fat diet on cognitive function and hippocampal gene expression in mice. Mol. Med. Rep..

[57] Hoizumi M., Sato T., Shimizu T., Kato S., Tsukiyama K., Narita T., Fujita H., Morii T., Sassa M.H., Seino Y. (2019). Inhibition of GIP signaling extends lifespan without caloric restriction. Biochem. Biophys. Res. Commun..

[58] Kanoski S.E., Fortin S.M., Arnold M., Grill H.J., Hayes M.R. (2011). Peripheral and central GLP-1 receptor populations mediate the anorectic effects of peripherally administered GLP-1 receptor agonists, liraglutide and exendin-4. Endocrinology.

[59] O’Neill H.M. (2013). AMPK and exercise: Glucose uptake and insulin sensitivity. Diabetes Metab. J..

[60] Timón-Gómez A., Nývltová E., Abriata L.A., Vila A.J., Hosler J., Barrientos A. (2018). Mitochondrial cytochrome c oxidase biogenesis: Recent developments. Semin. Cell Dev. Biol..

[61] Knezovic A., Osmanovic-Barilar J., Curlin M., Hof P.R., Simic G., Riederer P., Salkovic-Petrisic M. (2015). Staging of cognitive deficits and neuropathological and ultrastructural changes in streptozotocin-induced rat model of Alzheimer’s disease. J. Neural Transm..

[62] Salkovic-Petrisic M., Knezovic A., Hoyer S., Riederer P. (2013). What have we learned from the streptozotocin-induced animal model of sporadic Alzheimer’s disease, about the therapeutic strategies in Alzheimer’s research. J. Neural Transm..

[63] Babic Perhoc A., Osmanovic Barilar J., Knezovic A., Farkas V., Bagaric R., Svarc A., Grünblatt E., Riederer P., Salkovic-Petrisic M. (2019). Cognitive, behavioral and metabolic effects of oral galactose treatment in the transgenic Tg2576 mice. Neuropharmacology.

[64] Markaki I., Winther K., Catrina S.B., Svenningsson P. (2020). Repurposing GLP1 agonists for neurodegenerative diseases. Int. Rev. Neurobiol..

[65] Carlessi R., Chen Y., Rowlands J., Cruzat V.F., Keane K.N., Egan L., Mamotte C., Stokes R., Gunton J.E., de Bittencourt P.I.H. (2017). GLP-1 receptor signalling promotes β-cell glucose metabolism via mTOR-dependent HIF-1α activation. Sci. Rep..

[66] Fu Y., Kaneko K., Lin H.Y., Mo Q., Xu Y., Suganami T., Ravn P., Fukuda M. (2020). Gut Hormone GIP Induces Inflammation and Insulin Resistance in the Hypothalamus. Endocrinology.

[67] Noble E.P., Wurtman R.J., Axelrod J. (1967). A simple and rapid method for injecting H3-norepinephrine into the lateral ventricle of the rat brain. Life Sci..

[68] Osmanovic Barilar J., Knezovic A., Grünblatt E., Riederer P., Salkovic-Petrisic M. (2015). Nine-month follow-up of the insulin receptor signalling cascade in the brain of streptozotocin rat model of sporadic Alzheimer’s disease. J. Neural Transm..

[69] Kolarić Đ. (2021). Učinak Antagonista Receptora Glukagonu Sličnog Peptida-1 na Biokemijske Promjene u Mozgu Štakora. Ph.D. Thesis.

[70] Ng C.M., Tang F., Seeholzer S.H., Zou Y., De León D.D. (2018). Population pharmacokinetics of exendin-(9-39) and clinical dose selection in patients with congenital hyperinsulinism. Br. J. Clin. Pharmacol..

[71] Chopra A. (2004–2013). N-2-(4-[18F]-Fluorobenzamido)ethylmaleimide coupled to cysteine-tagged on the C- or N-terminal of exendin-4. Molecular Imaging and Contrast Agent Database (MICAD).

