# Supplementary material for: Divergent Effect of Central Incretin Receptors Inhibition in a Rat Model of Sporadic Alzheimer’s Disease
Source: Int J Mol Sci. 2022 Jan 4;23(1):548. doi: 10.3390/ijms23010548 (PMC8745186; doi:10.3390/ijms23010548)
Supplement: Supplementary file 1 [file ijms-23-00548-s001.zip › ijms-1501453 supplementary for pub.pdf]

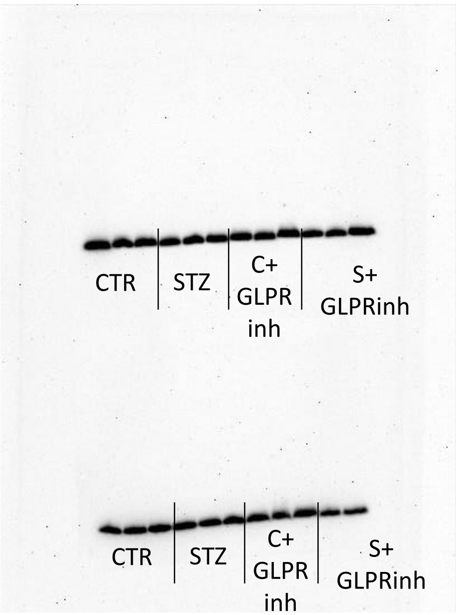

CytC – HPC
Figure 2Aiii

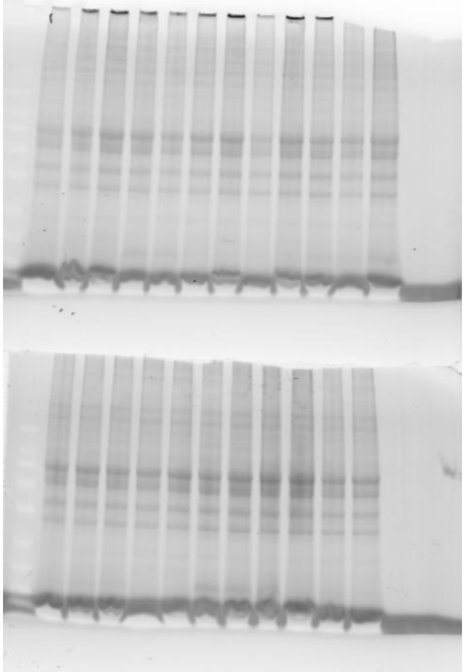

Total proteins on gels visualized using UV (TGX Stain-Free 12% gels) – Figure 2Aiii

COXIV – HPC
Figure 2Ai

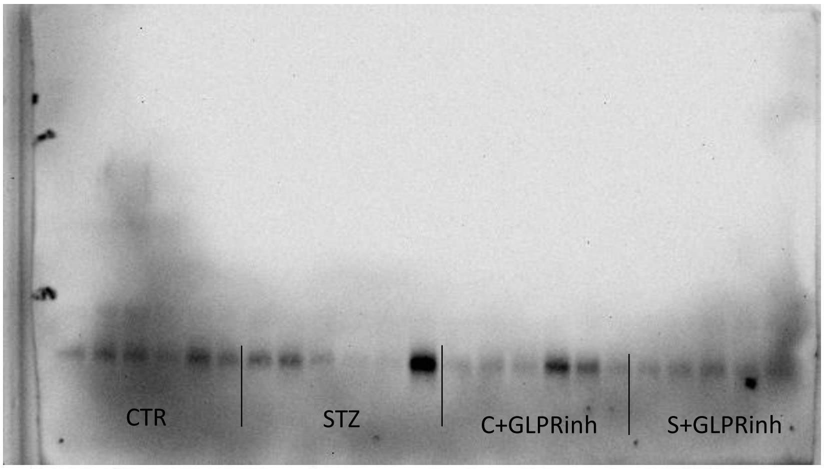

PDH – HPC
Figure 2Av

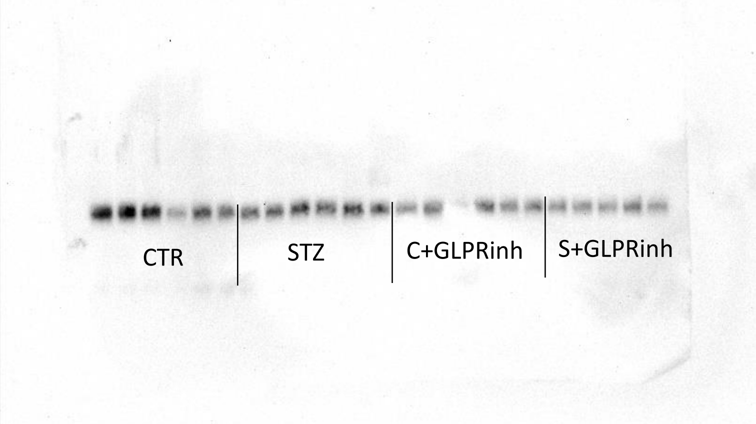

Total proteins on gels visualized using UV (TGX Stain-Free 12% gels) – For Figures 2Ai and 2Av

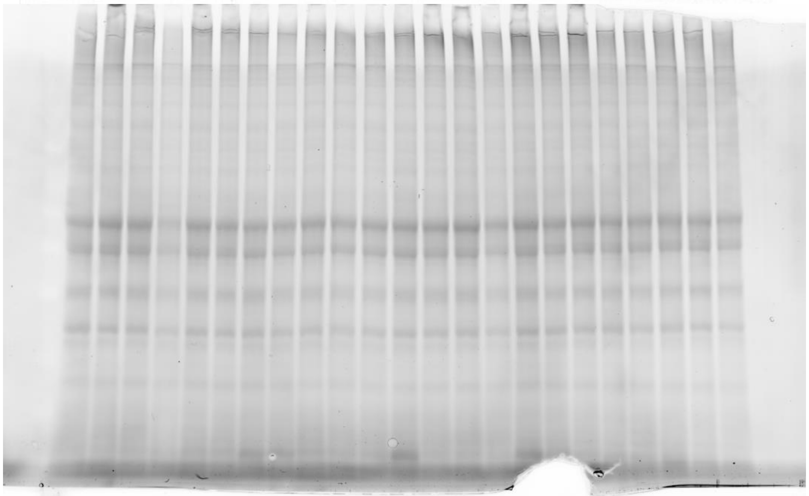

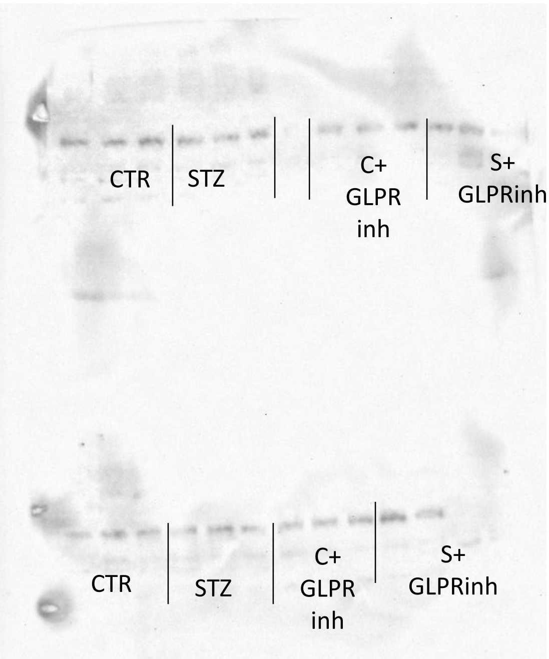

AMPK– HPC
Figure 3Aiii

Total proteins on gels visualized using UV (TGX Stain-Free 12% gels) – Figure 3Aiii

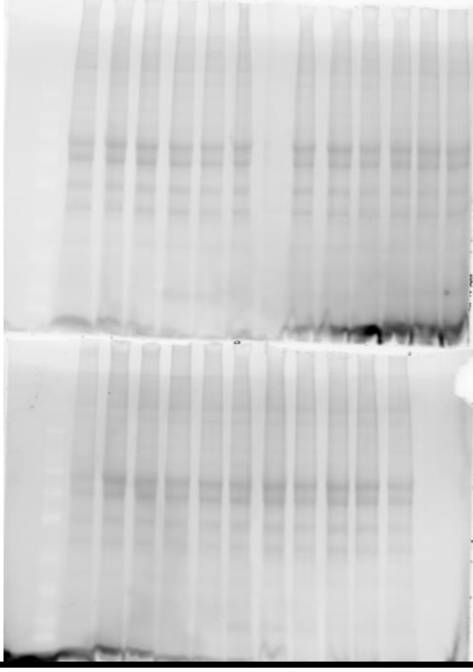

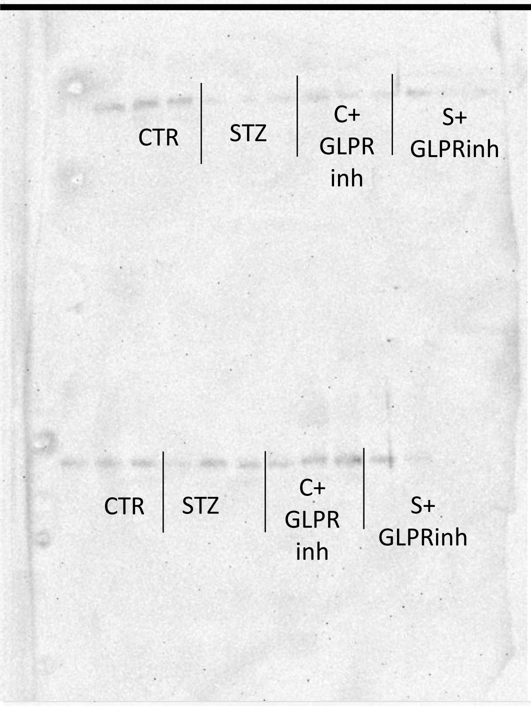

pAMPK– HPC
Figure 3Ai

Total proteins on gels visualized using UV (TGX Stain-Free 12% gels) – Figure 3Ai

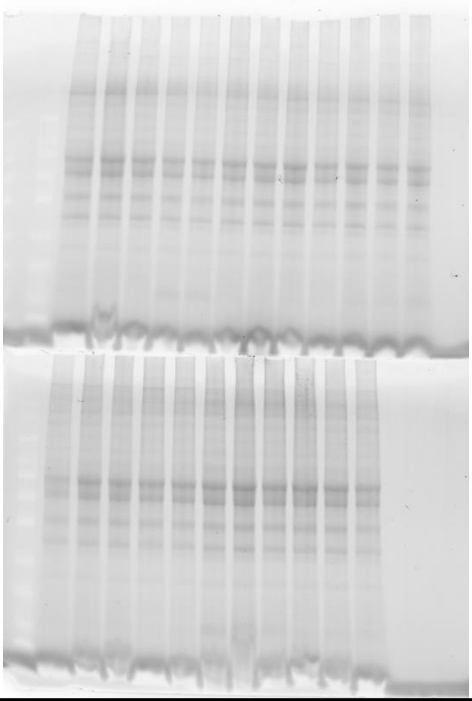

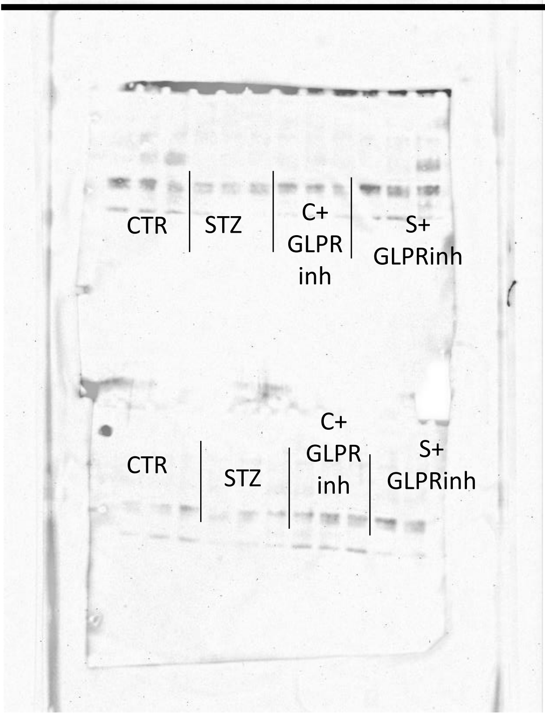

c-fos– HPC
Figure 4Ai

Total proteins on gels visualized using UV (TGX Stain-Free 12% gels) – Figure 4Ai

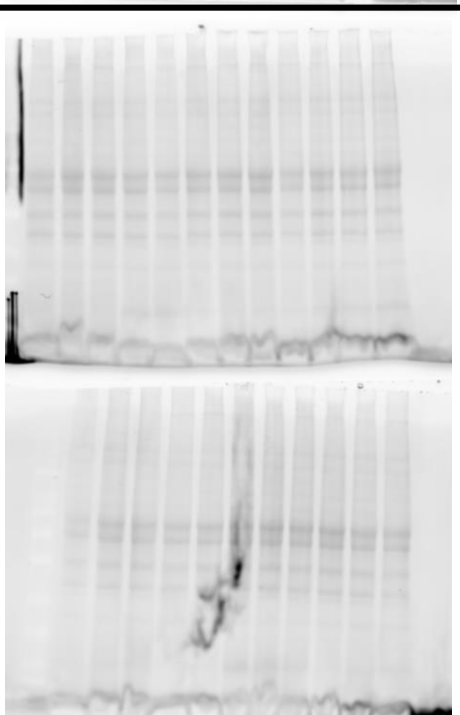

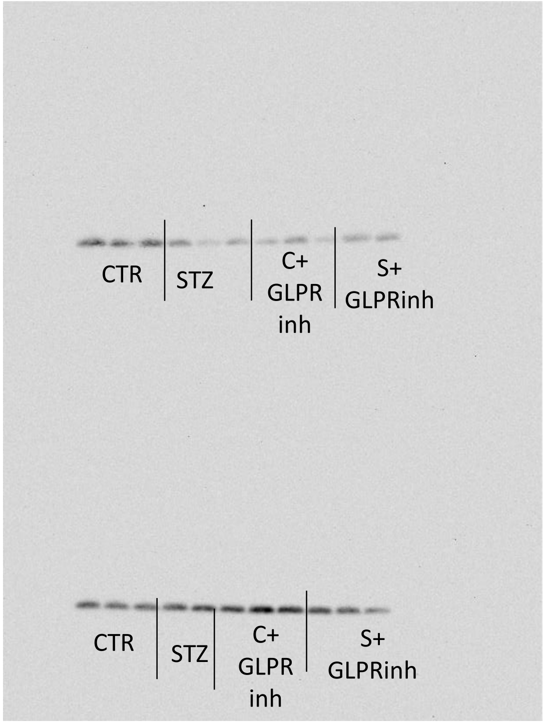

CytC– HPT
Figure 2Aiv

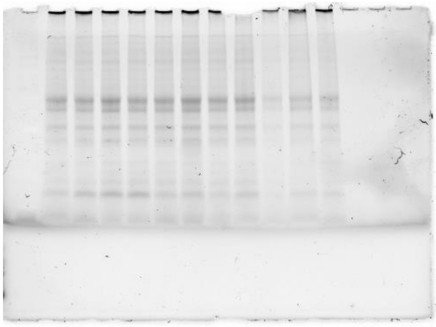

Total proteins on gels visualized using UV (TGX Stain-Free 12% gels) – Figure 2Aiv

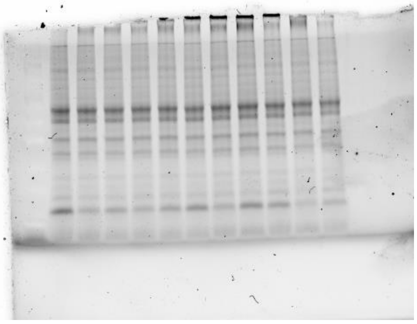

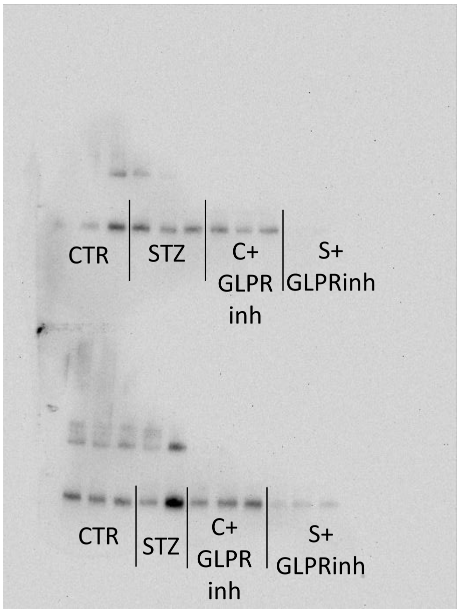

COXIV– HPT
Figure 2Aii

Total proteins on gels visualized using UV (TGX Stain-Free 12% gels) – Figure 2Aii

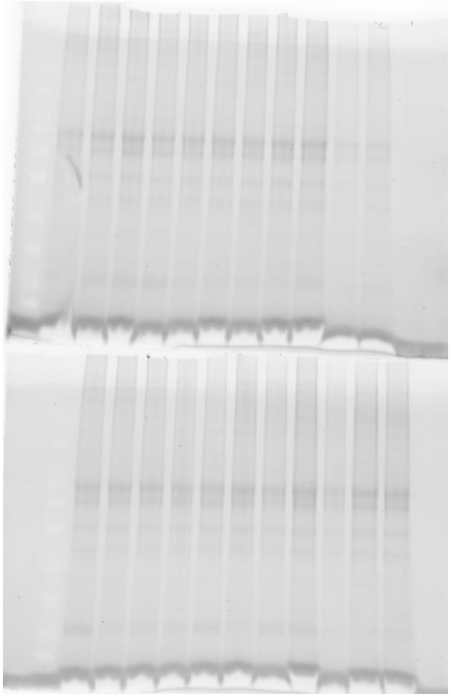

PDH– HPT - Figure 2Avi

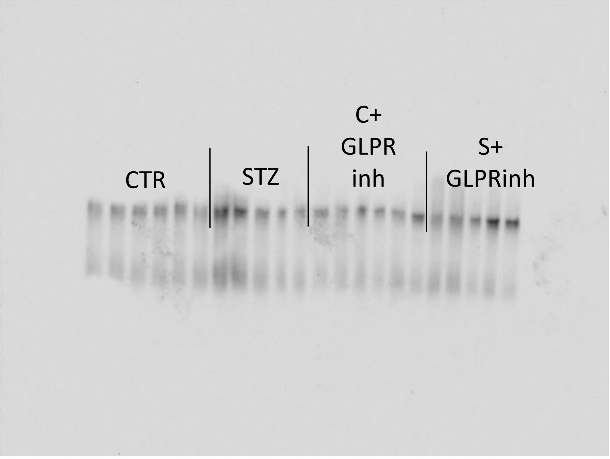

Total proteins on gels visualized using UV (TGX Stain-Free 12% gels) – Figure 2Avi

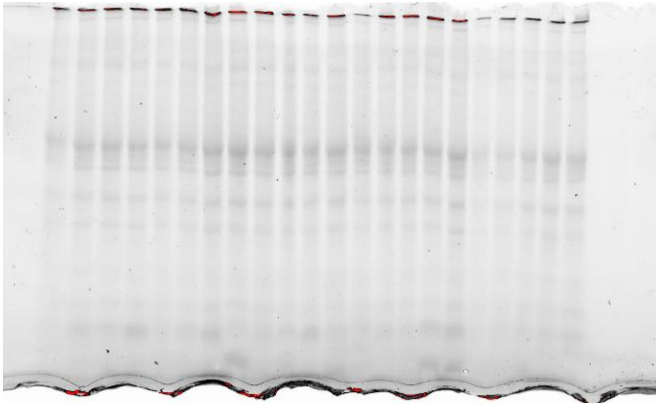

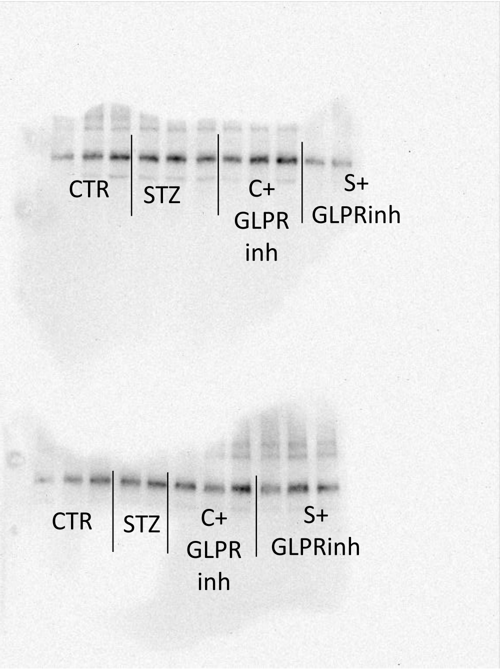

AMPK– HPT
Figure 3Aiv

Total proteins on gels visualized using UV (TGX Stain-Free 12% gels) – Figure 3Aiv

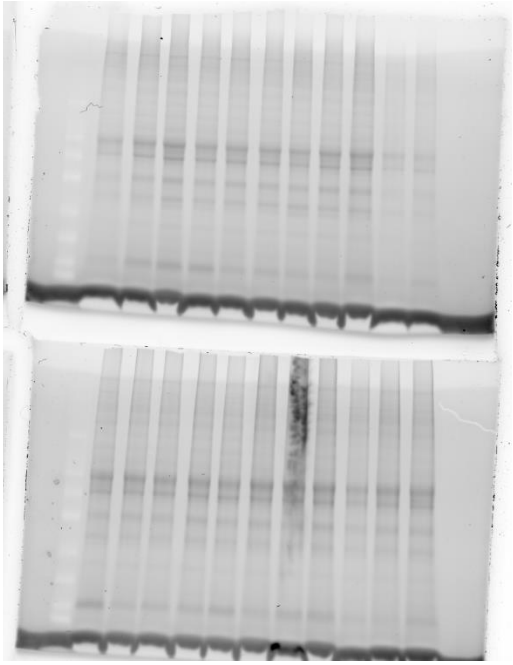

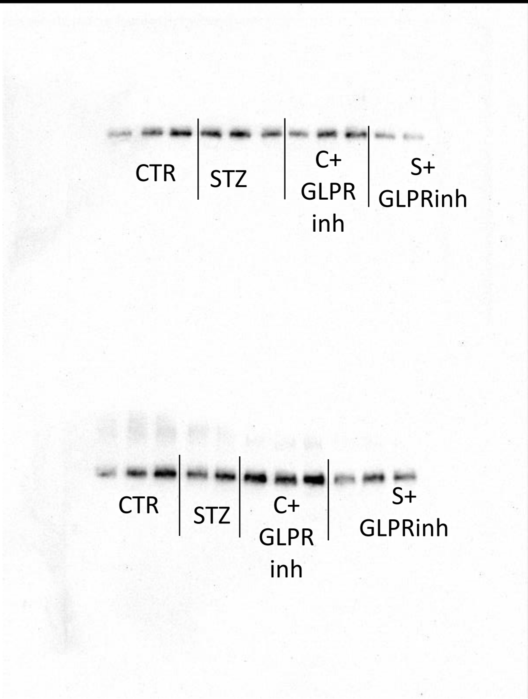

pAMPK– HPT
Figure 3Aii

Total proteins on gels visualized using UV (TGX Stain-Free 12% gels) – Figure 3Aii

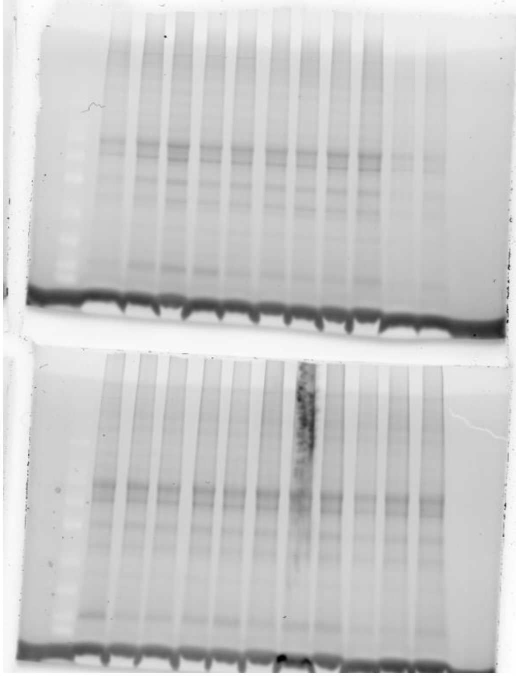

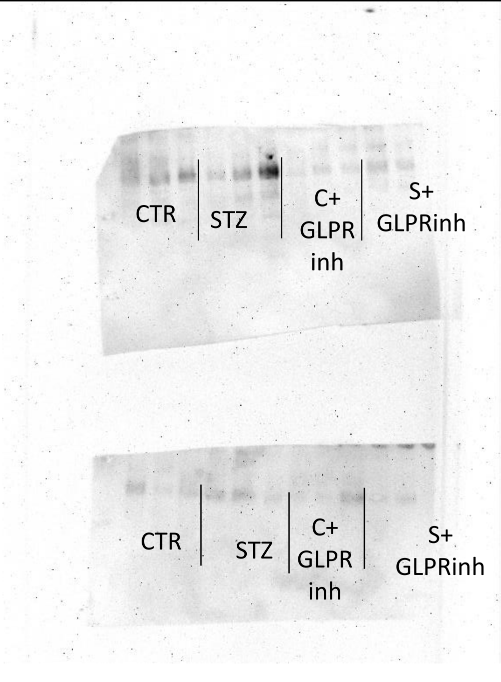

c-fos– HPT
Figure 4Aii

Total proteins on gels visualized using UV (TGX Stain-Free 12% gels) – Figure 4Aii

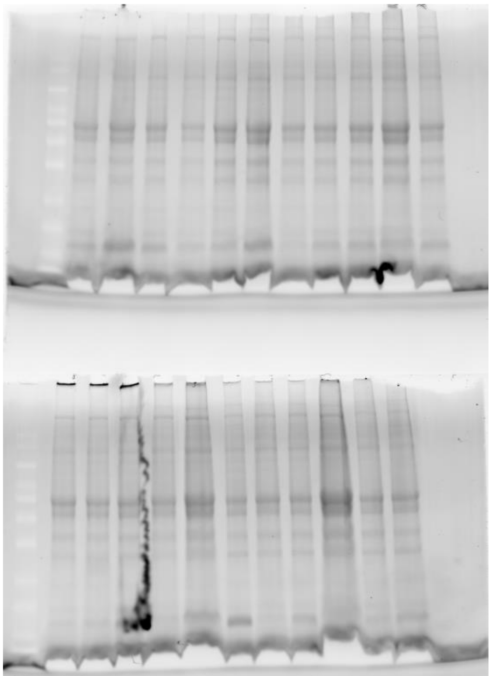

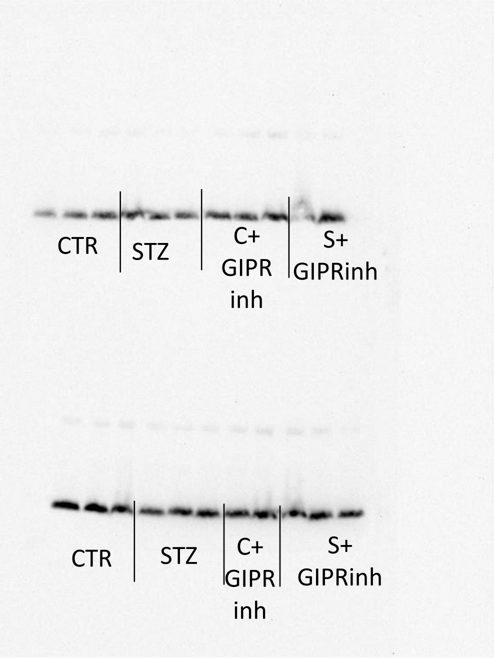

CytC– HPC
Figure 2Biii

Total proteins on gels visualized using UV (TGX Stain-Free 12% gels) – Figure 2Biii

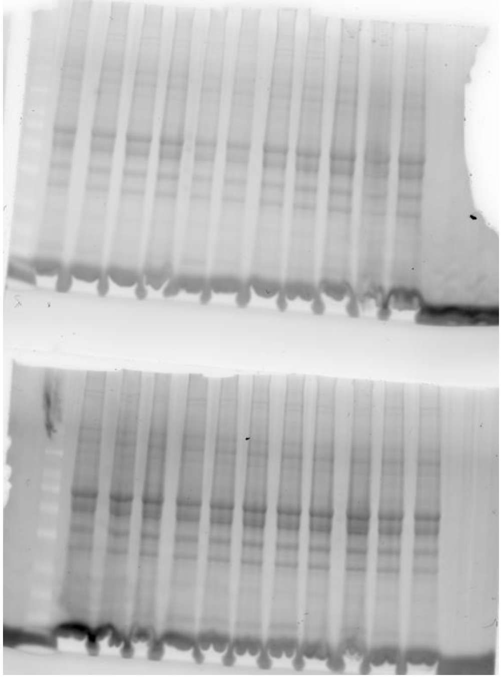

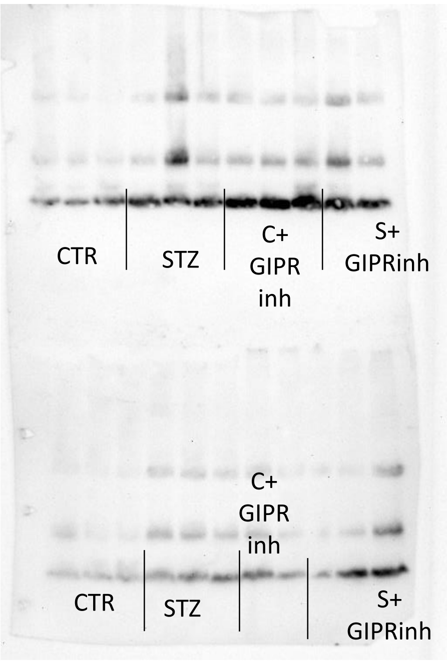

COXIV– HPC
Figure 2Bi

Total proteins on gels visualized using UV (TGX Stain-Free 12% gels) – Figure 2Bi

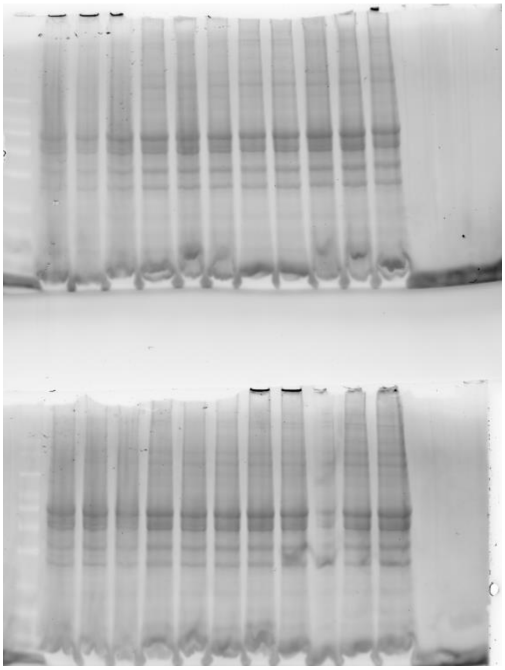

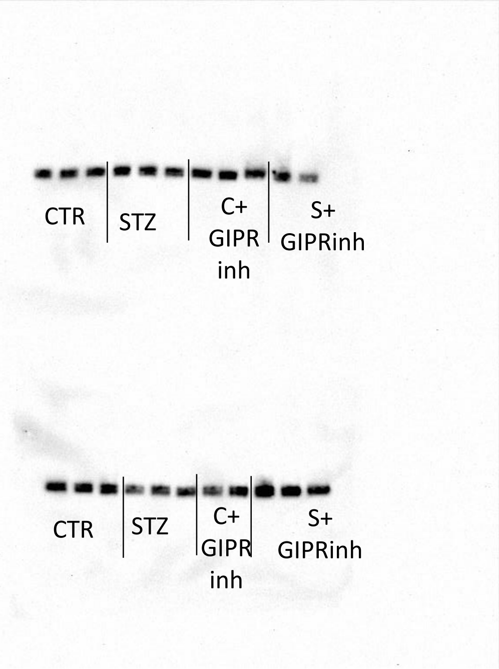

PDH– HPC
Figure 2Bv

Total proteins on gels visualized using UV (TGX Stain-Free 12% gels) – Figure 2Bv

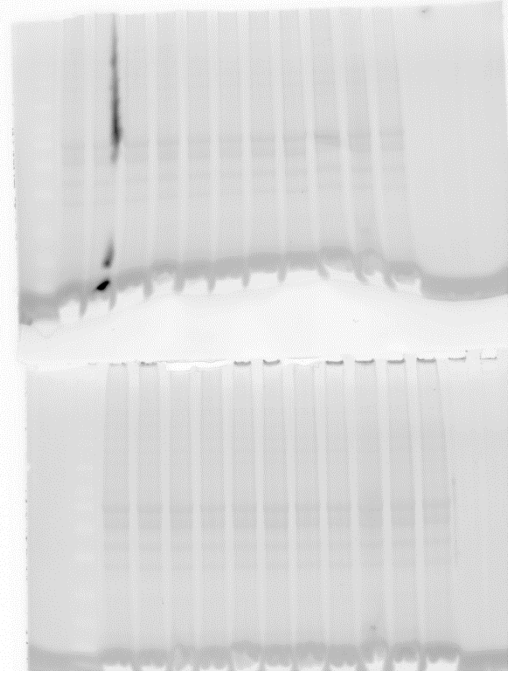

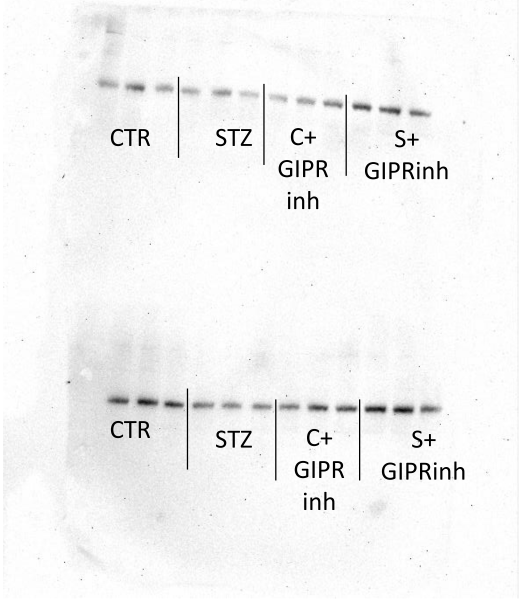

AMPK– HPC
Figure 3Biii

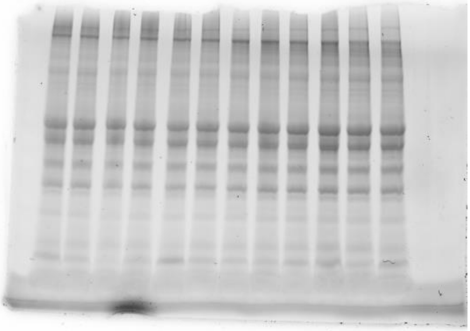

Total proteins on gels visualized using UV (TGX Stain-Free 12% gels) – Figure 3Biii

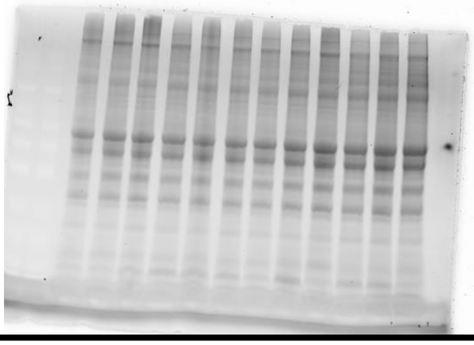

pAMPK and cAMP – HPC
Figure 3Bi and 4Biii

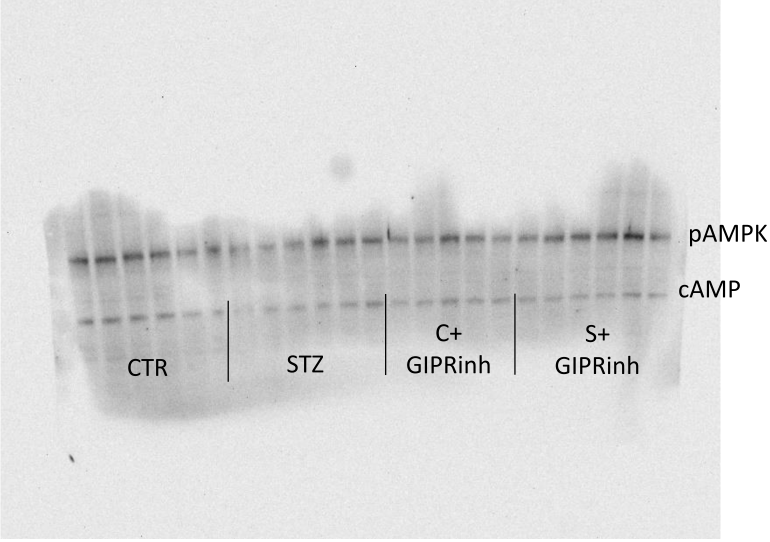

Total proteins on gels visualized using UV (TGX Stain-Free 12% gels) – Figure 3Bi and 4Biii

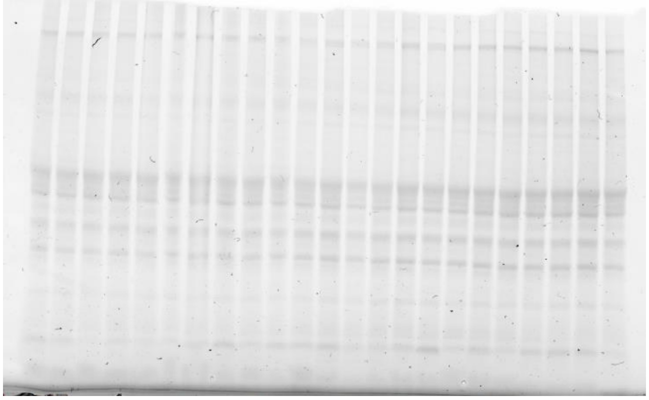

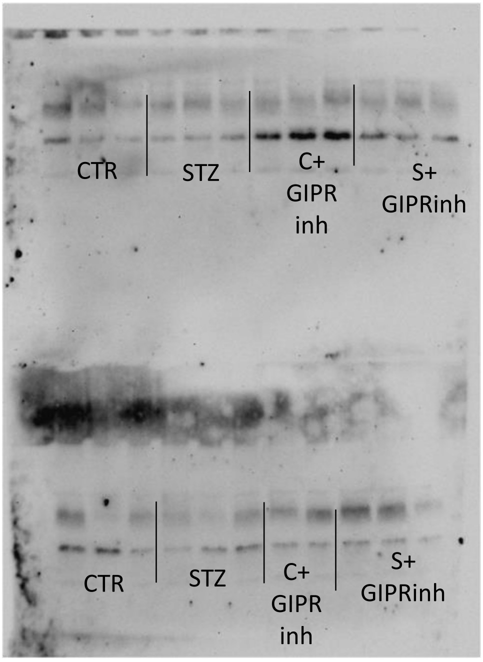

c-fos– HPC
Figure 4Bi

Total proteins on gels visualized using UV (TGX Stain-Free 12% gels) – Figure 4Bi

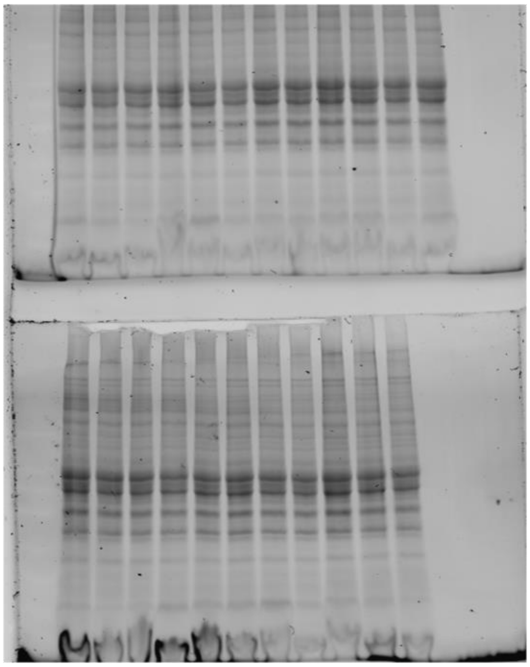

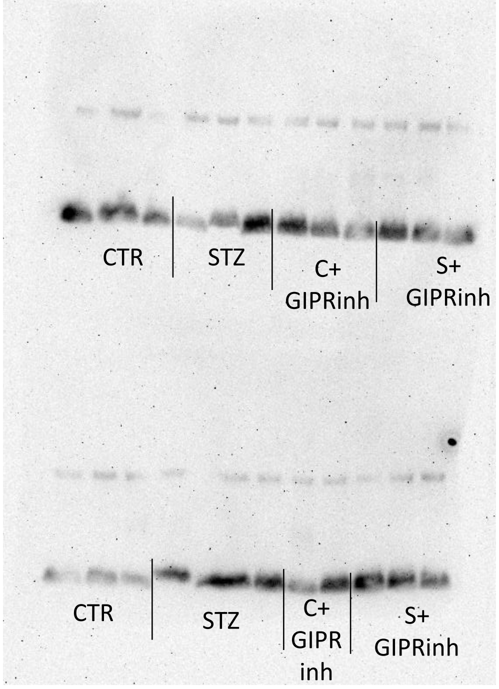

CytC– HPT
Figure 2Bvi

Total proteins on gels visualized using UV (TGX Stain-Free 12% gels) – Figure 2Bvi

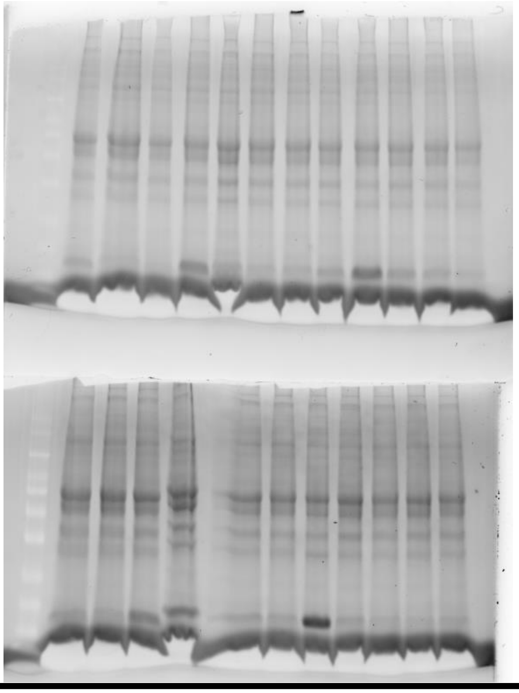

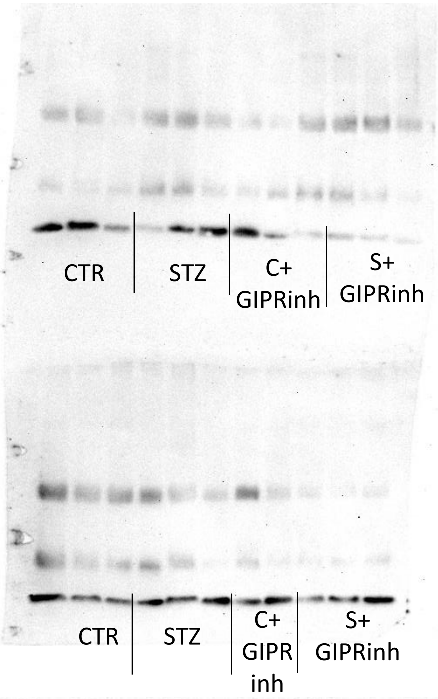

COXIV– HPT
Figure 2Bii

Total proteins on gels visualized using UV (TGX Stain-Free 12% gels) – Figure 2Bii

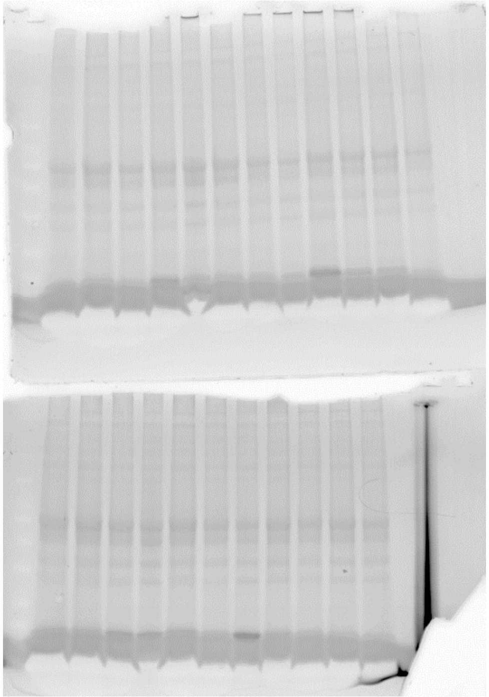

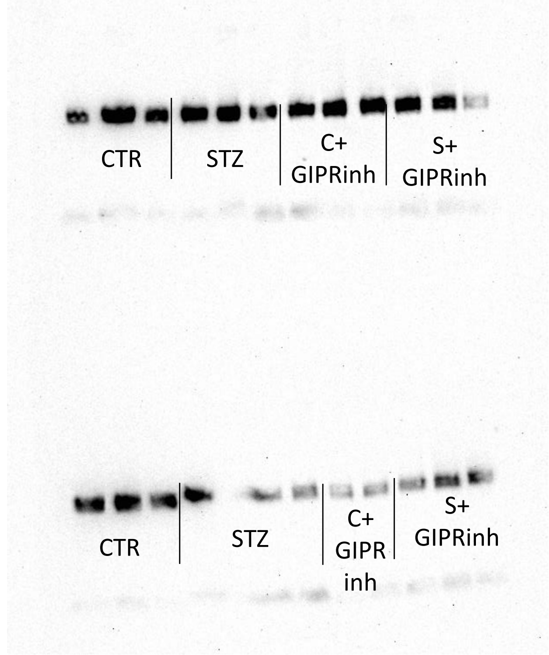

PDH – HPT
Figure 2Bvi

Total proteins on gels visualized using UV (TGX Stain-Free 12% gels) – Figure 2Bvi

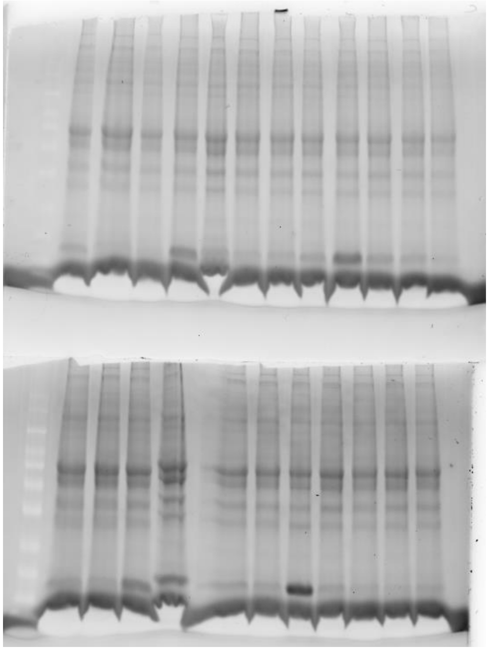

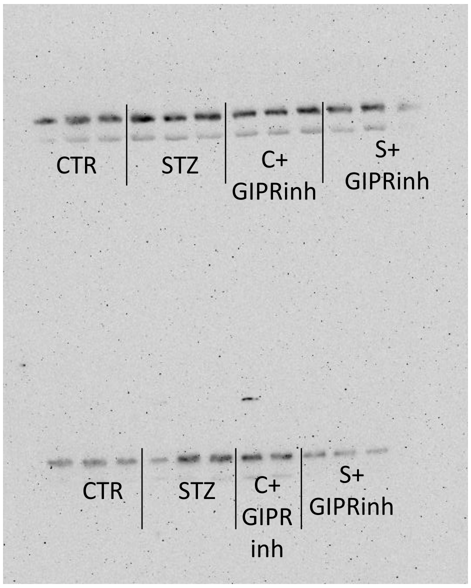

AMPK – HPT
Figure 3Bvi

Total proteins on gels visualized using UV (TGX Stain-Free 12% gels) – Figure 3Bvi

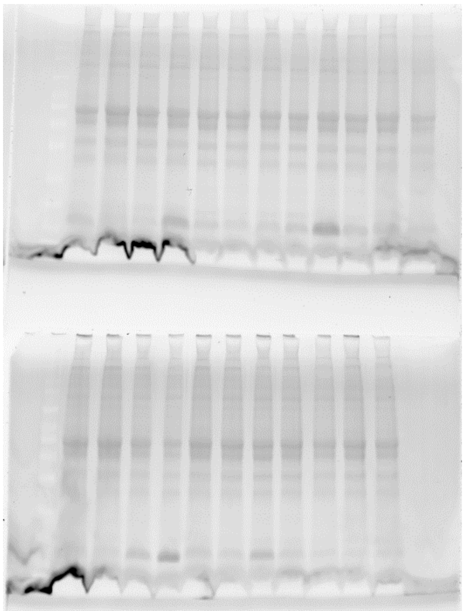

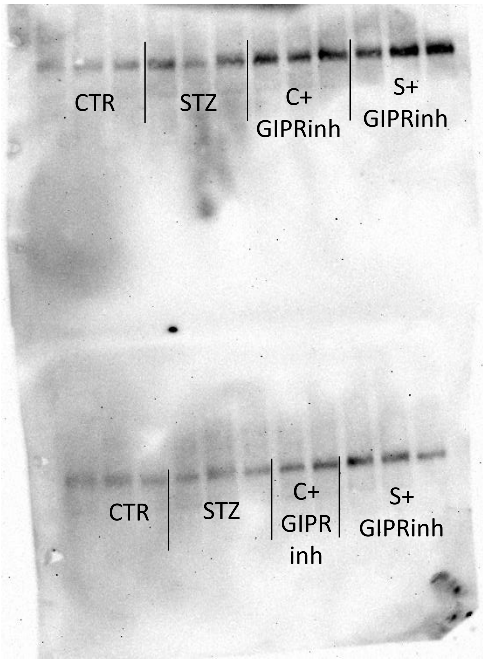

pAMPK – HPT
Figure 3Bii

Total proteins on gels visualized using UV (TGX Stain-Free 12% gels) – Figure 3Bii

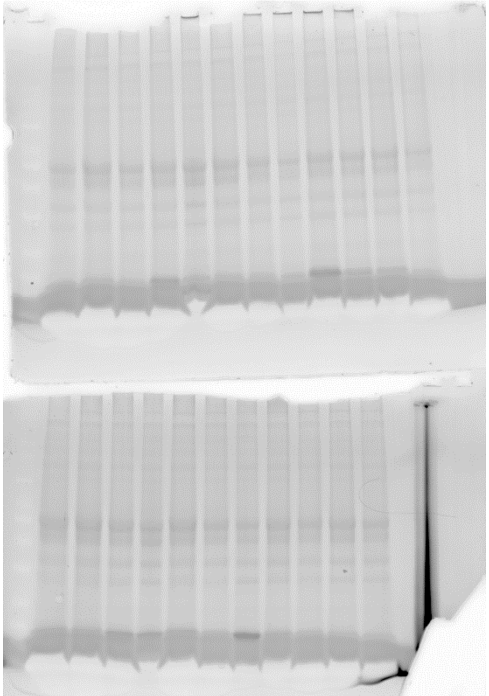

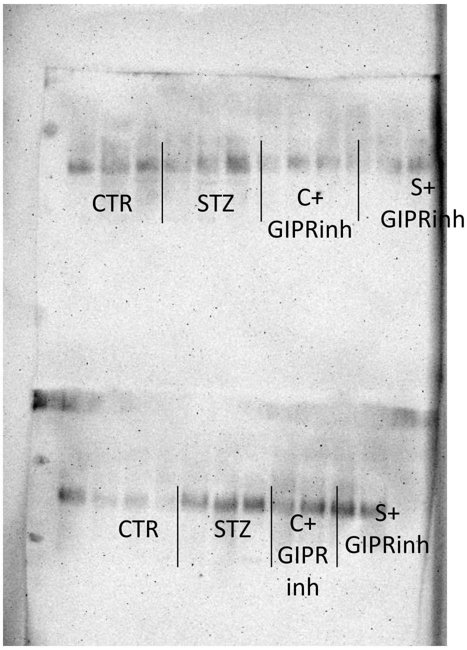

c-fos – HPT
Figure 4Bii

Total proteins on gels visualized using UV (TGX Stain-Free 12% gels) – Figure 4Bii

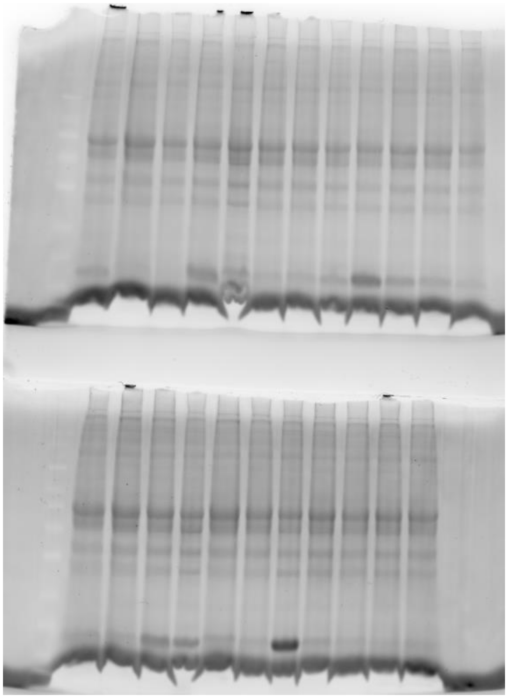

Group	Animal ID	Glucose-plasma	Insulin-plasma	GLP1total-plasma	GLP1active-plasma	GIPtotal-plasma	GIPactive-plasma	COXIV-HPC	CytC-HPC	PDH-HPC	pAMPK-HPC	tAMPK-HPC	p/tAMPK-HPC	cAMP-HPC	ATP-HPC	c-fos-HPC	COXIV-HPT	CytC-HPT	PDH-HPT	pAMPK-HPT	tAMPK-HPT	p/tAMPK-HPT	cAMP-HPT	ATP-HPT	c-fos-HPT
CTR	1	24.15113	1.162705	29.58177	1.983729	68.51255	5.054664	0.20404	2.98041	1.16577	0.45359	0.20991	2.160878	0.147436	14.89401	3.99073	0.69635	3.90971	0.82389	1.12478	1.85627	0.60594	0.237798	8.071765	2.01272
CTR	2	22.34757	1.489226	29.58177	9.575679	40.88034	5.433537	0.33356	2.3502	1.0322	0.28622	0.18842	1.519053	0.031234	16.68509	3.20304	0.41691	2.87981	0.67262	1.02617	1.8938	0.54186	0.432982	7.310697	1.33641
CTR	3	12.50111	4.566202	7.697762	3.215923	45.56278	4.761269	0.31084	1.80551	0.95749	0.3831	0.23643	1.620353		17.6297	2.33714	1.09318	2.84866	0.70224	1.08875	1.96995	0.55268	0.095953	8.66708	1.81666
CTR	4	19.45204	1.212482	37.97016	6.059312	92.44507	11.32224	0.36164	1.28747	0.66839	0.28763	0.19378	1.484312	0.212111	19.70155	1.41883	1.55367	1.96305	0.80676		0.68889	0.69429	0.348091	8.770143	1.56292
CTR	5	19.98668	3.27561	12.71329	4.234987	93.48561	7.180048	0.30891	1.46632	0.89821	0.20451	0.14543	1.406244	0.178998	29.84337	1.1404	1.61139	2.00693	1.11409	0.86982	1.13539	0.7661	0.222651	8.81706	0.86144
CTR	6	19.86754	0.7996929	12.48958	2.938238	69.14845	5.729242	0.21526	1.62939	0.70933	0.26834	0.13707	1.957686	0.022398	38.01725	1.90729	1.52644	2.16923	0.72009	1.28176	1.74118	0.73614	0.211378	9.194029	0.88427
CTR	7	19.70765	3.878794	17.57777	8.147182	102.0412	10.84634																		
CTR	8	19.27538	1.864449	14.38193	10.03734	53.13563	8.328218																		
CTR	9		1.146293	18.28394	3.345893	38.22116	3.358977																		
CTR	10	21.18072	2.575054	15.46092	3.665951	64.81285	5.207137																		
STZ	11	20.19688	0.9067629	10.5231	15.06904	36.19789	4.273816	0.32961	1.72851	0.64639	0.33134	0.18186	1.821951	0.108696		1.93479	1.10049	1.71112	1.46488	1.1553	2.07291	0.557332	0.020398	9.48816	1.21948
STZ	12	21.74773	1.868578	12.07221	5.749197	82.55989	11.52784	0.40943	2.44338	0.72688	0.33592	0.18719	1.79454	0.106093		1.79415	0.70623	0.5083	1.28313	1.29054	2.21521	0.582581	0.177762	8.97692	1.37202
STZ	13	19.70765	0.7879735	12.22481	12.48207	68.62817	5.907127	0.25331	2.09679	0.85771	0.28948	0.20862	1.387595	0.09333	19.6215	2.16043	0.72578	2.58492	0.74234	0.97157	1.93914	0.501031	0.126593	7.62096	2.39652
STZ	14	20.7008	0.5916355	20.44581		96.43382	7.947034	0.19111	1.60352	0.83478	0.23882	0.12477	1.914082	0.094961	16.08115	1.19135	1.22427	2.7066	0.55816	0.75968	1.47871	0.513745	0.01657	7.075745	1.37378
STZ	15	18.80639	5.697976	8.436341	9.153773	130.3671	13.73178	0.15436	1.29462	0.9189	0.25749	0.17361	1.483152	0.114891	20.83353	0.89331		2.79662	0.78921				0.467268	8.415032	1.00045
STZ	16	20.55986	2.682752		10.7226	245.5785	17.79542		1.25808	0.57059	0.21894	0.09282	2.358759	0.090153	18.66938	0.95572	3.2322			1.07871	1.73747	0.620851	0.432982	8.929849	0.98521
STZ	17	18.29843	1.593895	14.28418	19.49201		2.453379																		
STZ	18	16.92793	1.269418	43.47187	5.365859	247.0237	16.10435																		
STZ	19	19.93381	1.66064	13.37785	11.51805	52.73097	4.188339																		
STZ	20	20.92946	4.458108	8.974483	25.91761	59.61013	5.327267																		
CTR+GLP1Rinh	21	17.79283	1.898046	9.325338	10.77987	92.21383	4.837505	0.1652	1.82246	0.34043	0.26122	0.14884	1.755039	0.135205	25.39805	2.66375	0.89725	0.8227	0.80345	0.71043	1.62555	0.43704	0.185307	9.67884	0.61005
CTR+GLP1Rinh	22	21.35487	1.151765	8.082948	2.350631	133.8934	12.27866	0.17061	2.43148	0.42739	0.23289	0.12621	1.845258	0.077676	17.27034	2.28229	0.54025	0.80955	0.85699	1.02672	2.00563	0.511919	0.098991	9.745749	0.56937
CTR+GLP1Rinh	23	19.76108	0.7416591	18.31255		85.21907	5.00846	0.19932	1.7309	0.08051	0.35379	0.15161	2.333553	0.166439	37.54315	2.24944	0.52083	1.48375	0.93496	0.89476	1.80677	0.495226	0.241953	9.421592	0.41754
CTR+GLP1Rinh	24	19.06973	1.989157	14.90243	2.607749	111.7529	7.750667	0.5196	0.99476	0.39092	0.19423		2.133692	0.04222	36.46833	1.36586	1.16221	3.603	0.75759	1.27727	1.3748	0.929059	0.422608	8.136518	1.01147
CTR+GLP1Rinh	25	18.1408	1.250656	14.57876	8.49749375	98.51492	5.186345	0.44034	0.80201	0.42601	0.24027	0.1223	1.964595	0.057865	17.78442	2.17971	1.35794	2.84698	0.73689	0.92018	0.99847	0.92159	0.100025	7.776872	1.00622
CTR+GLP1Rinh	26	22.98867	1.212482	14.87737	2.990348	96.08697	5.137831	0.14802	0.8792	0.47334	0.32142	0.15171	2.118647	0.208469	20.61629	1.51627	1.30305	2.61839	0.85064	1.48497	1.61182	0.9213	0.127032	7.685096	0.86946
CTR+GLP1Rinh	27	19.30265	1.162098	14.43097	2.690446	110.0187	8.790257																		
CTR+GLP1Rinh	28	20.58555	0.7781002	17.21745	18.35677	20.70534	1.041846																		
CTR+GLP1Rinh	29	22.68798	0.5169137	15.53813	24.02078	42.73019	7.727565																		
CTR+GLP1Rinh	30	17.88035	3.378628	5.742137	4.183356	81.51935	10.2041																		
STZ+GLP1Rinh	31	21.62573	1.29421	8.035804	1.979355	94.0637	4.234543																		
STZ+GLP1Rinh	32	18.55391	1.904524	22.20505		64.69723	6.607119	0.24068	1.57056	0.4122	0.30489	0.21304	1.43114	0.187243	20.15718	5.40737	0.23917	1.34136	1.99274	0.77741	1.65727	0.469091	0.132424	8.071765	0.57764
STZ+GLP1Rinh	33				5.045563		4.463253	0.25031	1.77098	0.43017	0.20497	0.14166	1.446915	0.373061	20.18409	4.02184	0.14353	2.10258	2.06387	0.40367	1.28433	0.314304	0.077408	8.864024	0.702665
STZ+GLP1Rinh	34	19.85426	2.279997	59.54348	6.816654	162.5084	9.194542	0.35488	1.71528	0.4511	0.2124			0.102836	21.27033	4.59841	0.69017	1.74987	1.1638	0.75933	1.68413	0.450874	0.799369	15.70215	0.5089
STZ+GLP1Rinh	35	21.74773	5.725374	34.68185	3.64867	111.3483	8.092577	0.37085	0.9329	0.69415	0.20895	0.24203	0.863323	0.11569	11.31715	2.35231	0.36013	2.31664	1.55827	0.90362	1.58616	0.56969	0.056871	10.14905	0.97506
STZ+GLP1Rinh	36	19.68721	2.6800031	3.7513	2.525001	81.70002		0.53149	0.81077	0.79438	0.16687	0.16838	0.991032	0.094632	29.47315	1.96555	0.22751	1.25251	1.29468	0.62653	1.06963	0.585745			0.74906
STZ+GLP1Rinh	37	18.35547	2.722502	24.056872	4.497139	58.85862	2.679778																		
STZ+GLP1Rinh	38	19.22073	1.417184	11.30904	5.668362	57.58685	9.335464																		
STZ+GLP1Rinh	39	23.10321	2.061198	36.49778	3.536289	64.40819	4.969186																		
STZ+GLP1Rinh	40	15.03667	4.035036	16.43067	10.95972	40.12883	2.439517																		

Group	Animal ID	Glucose-plasma	Insulin-plasma	GLP1total-plasma	GLP1active-plasma	GIPtotal-plasma	GIPactive-plasma	COXIV-HPC	CytC-HPC	PDH-HPC	pAMPK-HPC	tAMPK-HPC	p/tAMPK-HPC	cAMP-HPC	ATP-HPC	c-fos-HPC	COXIV-HPT	CytC-HPT	PDH-HPT	pAMPK-HPT	tAMPK-HPT	p/tAMPK-HPT	cAMP-HPT	ATP-HPT	c-fos-HPT
CTR	1	16.15716	3.375331	14.54458	2.1563	172.6468	3.982318	0.30629	1.21848	5.62748	2.28864	3.20843	0.713321	1.67199	17.7328	0.71776	2.26891	1.42809	1.29691	0.56496	0.82782	0.682467	0.177147	16.78641	0.87934
CTR	2	0.505749	1.026209	10.57815	1.799	182.3813	1.930009	0.34012	1.77628	6.45173	2.58621	3.26968	0.790967	1.72187	17.70701	0.53616	2.04399	0.78274	2.10202	0.64737	0.91549	0.70713	0.300959	13.66298	0.77445
CTR	3	12.34426	4.139353	10.12048	2.5676	285.0963	1.433134	0.41937	2.11734	6.33279	2.6898	2.15733	1.246819	1.66207	18.59073	0.4612	1.14519	0.73096	1.7626	0.80881	0.95798	0.844287	0.029653	17.7328	1.32279
CTR	4	18.33046		7.902568	2.3534	1993.656	2.059628	0.21744	0.80556	6.70185	2.08492	6.07105	0.34342	1.46833	20.61629	0.63505	1.35861	0.16235	0.65437	0.29238	0.31544	0.926896	0.905536	19.0378	1.09267
CTR	5	13.75628	4.16679	9.475059	2.0312	1978.216	1.800389	0.19208	0.89948	6.02194	1.65936	4.84452	0.342523	1.55227	25.33977	0.91066	0.64174	0.17754	0.79866	0.30611	0.43532	0.703184	0.164719	18.64315	0.24746
CTR	6	15.2964	3.68612	11.09449	1.9772	477.4352	1.930009	0.2261	0.84901	6.23385	2.51178	3.45996	0.725956	1.43548	15.88387	0.5265	0.65525	0.15336	0.60969	0.3226	0.48683	0.662654	0.08441	23.27395	0.19167
CTR	7	24.0537	3.751459	19.94268	2.7449	111.2192	2.059628																		
CTR	8	10.61243	1.001754	9.580673	2.2193	182.0456	4.068731																		
CTR	9	20.58334	2.513842	14.77928	2.078	111.8905	2.059628																		
CTR	10	13.24417	3.884683	13.26546	2.9312	253.2076	1.54115																		
STZ	11	18.8217	2.645205	8.735753	1.7864	1043.039	2.362074	0.66922	2.13557	6.64072	1.49714	1.81611	0.824366	0.71154	28.85995	0.40806	0.54765	0.31229	1.8902	0.8515	1.36283	0.624803	0.352947	21.6275	0.78438
STZ	12	16.50362	0.867434	4.335128	1.56284	2307.844	3.053379	0.55675	2.19272	6.91686	1.30859	2.11284	0.619351	0.70282	18.64315	0.39705	1.15022	0.45641	1.7065	0.60043	1.18838	0.505251	0.163016	17.88782	0.62596
STZ	13	18.54234	0.893865	8.946983	1.7864	666.4174	1.951612	0.71317	1.80282	6.95927	1.59522	1.08901	1.464835	0.87765	19.72826	0.62567	1.31904	0.97104	1.02481	0.89727	1.37405	0.653011	0.287707	16.9642	1.08798
STZ	14	17.06281	0.815105	10.30824	1.9772	459.309	2.340471	0.22721	0.83798	3.76782	2.18685	2.60113	0.840731	1.00623	17.60395	0.45853	0.88432	0.2738	0.50313	0.29466		0.706754	0.507775	17.19365	0.1525
STZ	15	17.4091	0.925177	11.77512	2.375	126.66	2.858948	0.33388	1.0767	4.66911	1.90345	1.82005	1.045823	1.00018	19.19642	0.58064	0.53232	0.47073	0.1959	0.35561	0.77619	0.458148	0.1986	18.17313	0.42675
STZ	16	15.22634	8.971501	16.44565	2.1104	75.97386	1.865199	0.32027	1.09486	4.6343	1.744	1.79973	0.969034	1.08284	21.54489	0.7213	0.86109	0.44169	0.2143	0.3893	0.79962	0.486856	0.082674	17.55249	0.7283
STZ	17	0.7030201	5.416866	11.10622	1.9259	249.8509																			
STZ	18	12.36048	4.06687	5.731593	2.1932	2060.119	2.686123																		
STZ	19	26.17868	2.962806	6.024968	2.0195	1838.913	2.102835																		
STZ	20	14.54072	8.13975	11.29398	2.7584	1476.725	1.973216																		
CTR+GIPRinh	21	20.07588	9.80827	13.96956	2.7575	3839.506	513.8839	1.12782	1.69243	7.91797	1.95743	1.51031	1.296045	1.07921	24.4427	1.26716	1.72214	0.9747	1.48728	1.50967	1.14214	1.321791	0.302003	22.45946	0.31099
CTR+GIPRinh	22	30.70064	4.658765	8.841368	1.9484	119.6109	204.1796	1.17078	1.38191	7.76871	1.60512	1.20898	1.327665	0.67709	29.16595	1.86042	0.66165	0.72468	1.91676	1.74902	1.19807	1.459865	0.51308	17.14257	0.68214
CTR+GIPRinh	23	15.03968	4.183654	12.43228	2.1176	170.6328	763.963	1.18416	1.49602	7.49287	1.46062	1.55315	0.940424	0.94836	27.34795	1.69186	0.21332	0.55147	1.73015	1.43543	1.11345	1.289173	0.017333	19.80845	0.77743
CTR+GIPRinh	24	15.05425	8.517301	13.19505	2.294	2313.886	838.9911	0.40834	1.27246	5.04308	1.24017	2.06791	0.599721	0.76572	18.17313	0.57071	0.54314	0.22433	0.12814	0.46019	0.78604	0.585454	0.100025	19.8352	0.81718
CTR+GIPRinh	25	25.01343	8.748527	17.43139	2.3633	4157.05	191.3689	0.23526	1.36786	5.62154	0.93372	1.53328	0.608969	0.8094	16.03363	0.54006	0.70594	0.3841	0.14433	0.52451	0.62982	0.832793	0.269381	12.78941	0.75771
CTR+GIPRinh	26	16.8091	3.513341	9.568938	2.3291	2263.2	855.2584																		
CTR+GIPRinh	27	11.73835	5.661367	6.212727		4012.04	80.84668																		
CTR+GIPRinh	28	12.94242	8.359073	11.81032	2.6342	2020.846	430.9489																		
STZ+GIPRinh	29	16.52353	6.299046	13.35934	2.7395	2680.102	129.2812	0.75277	0.90645	7.07201	0.9315	1.83944	0.506404	0.57437	20.15718	0.89172	0.32908	0.89955	1.49764	1.36467	0.89943	1.517261	0.346887	17.52678	0.59996
STZ+GIPRinh	30	37.42585	9.337687	9.181682	2.2256	2938.232	119.6677	0.60063	1.39082	6.08089	1.15839	2.34612	0.493747	0.66963	18.38155	0.75319	0.15421	0.67796	1.26015	1.76014	0.78422	2.244447	0.254857	20.50795	0.77595
STZ+GIPRinh	31	12.18244	7.294388	9.181682	2.2904	1519.019	239.825	0.36834	1.11113	6.74139	0.91181	1.59753	0.570762	0.52914	22.48737	0.59852	0.1519	0.6944	0.3199	2.37542	0.2807		0.349298	17.86195	1.52897
STZ+GIPRinh	32	15.90218	6.535737	3.478472	1.7666	3077.871	215.6726	0.61495	1.11281	5.54052	0.96535	2.35773	0.40944	0.84283	24.41395	0.50524	0.33241	0.51683	0.22737	0.71701	0.49499	1.448534	0.163016	14.77194	0.94229
STZ+GIPRinh	33	12.15337	6.569736	4.921877	2.0573	2182.974	289.0804	0.65378	1.0806	5.68357	1.28222	2.18765	0.586118	0.80069	28.67693	0.55699	0.47544	0.39487	0.39676	0.58044	0.30758	1.887119		22.45946	1.08856
STZ+GIPRinh	34	5.126925	1.526362	7.069383	3.977	2105.099	51.0342				0.6977	1.32045	0.52838	0.85429		0.37789	0.79474	0.27208	0.27755	0.46538	0.2024	2.299308	0.139004	20.72481	0.73294
STZ+GIPRinh	35	24.12253	6.113601	5.555568	2.0654	2382.698	368.3427																		
STZ+GIPRinh	36	32.60823	7.940767	8.888308	3.347	2443.79	446.2008																		
STZ+GIPRinh	37	8.887614	6.568378	7.069383	2.3147	2364.908	115.6927																		
STZ+GIPRinh	38	16.24307	3.484942	19.97788	2.8565	2771.068	286.9417																		

49 Spadina Ave. Suite 200
Toronto ON M5V 2J1 Canada
www.biorender.com

Confirmation of Publication and Licensing Rights

December 27th, 2021
Science Suite Inc.

Subscription:	*Student Plan*
Agreement number:	*FQ23D8RUNI*
Journal name:	*International Journal of Molecular Sciences*

To whom this may concern,

This document is to confirm that Anthony Farma has been granted a license to use the BioRender content, including icons, templates and other original artwork, appearing in the attached completed graphic pursuant to BioRender's Academic License Terms. This license permits BioRender content to be sublicensed for use in journal publications.

All rights and ownership of BioRender content are reserved by BioRender. All completed graphics must be accompanied by the following citation: “Created with BioRender.com”.

BioRender content included in the completed graphic is not licensed for any commercial uses beyond publication in a journal. For any commercial use of this figure, users may, if allowed, recreate it in BioRender under an Industry BioRender Plan.

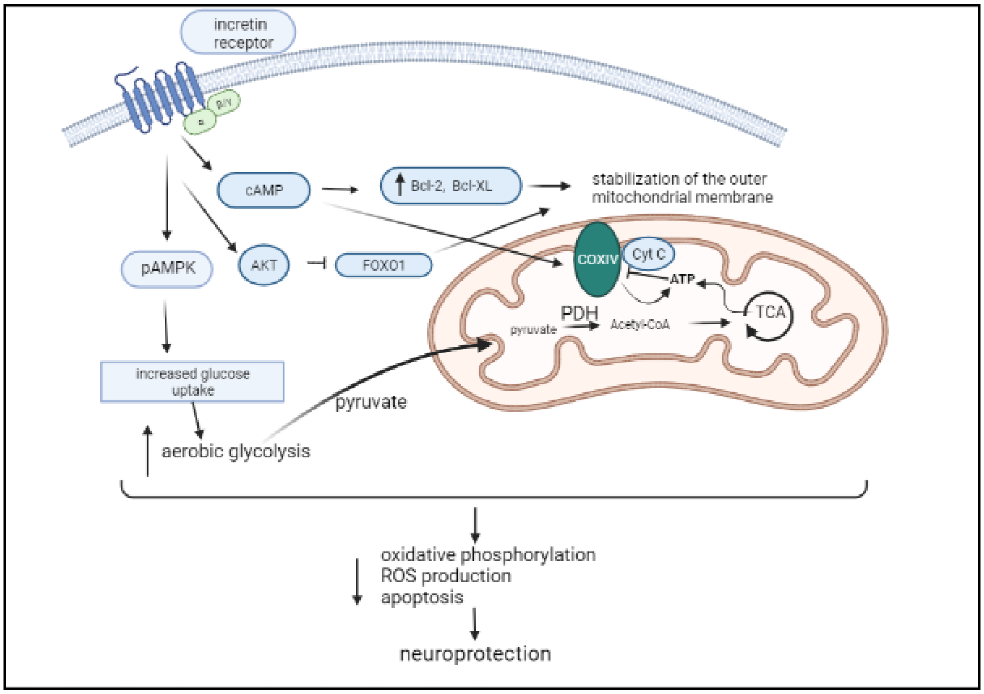

For any questions regarding this document, or other questions about publishing with BioRender refer to our BioRender Publication Guide, or contact BioRender Support at support@biorender.com.

49 Spadina Ave. Suite 200
Toronto ON M5V 2J1 Canada
www.biorender.com

Confirmation of Publication and Licensing Rights

December 21st, 2021
Science Suite Inc.

Subscription: *Student Plan*
Agreement number: *WA23CFFHT9*
Journal name: *international journal of molecular sciences*

To whom this may concern,

This document is to confirm that Anthony Farma has been granted a license to use the BioRender content, including icons, templates and other original artwork, appearing in the attached completed graphic pursuant to BioRender's Academic License Terms. This license permits BioRender content to be sublicensed for use in journal publications.

All rights and ownership of BioRender content are reserved by BioRender. All completed graphics must be accompanied by the following citation: “Created with BioRender.com”.

BioRender content included in the completed graphic is not licensed for any commercial uses beyond publication in a journal. For any commercial use of this figure, users may, if allowed, recreate it in BioRender under an Industry BioRender Plan.

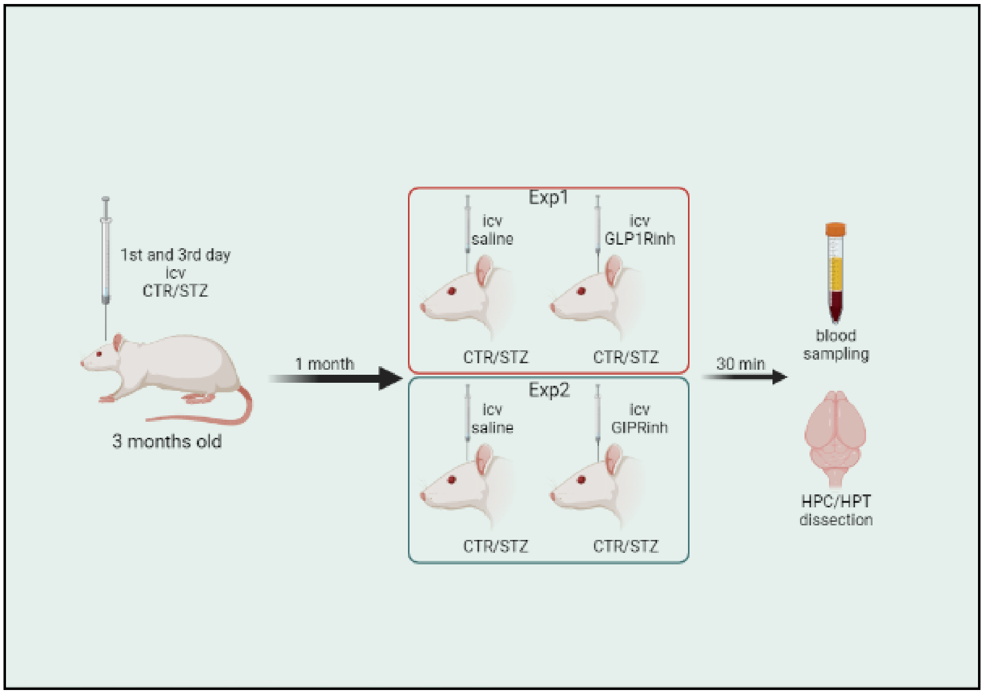

For any questions regarding this document, or other questions about publishing with BioRender refer to our BioRender Publication Guide, or contact BioRender Support at support@biorender.com.
